# Dual Thermal- and Oxidation-Responsive Polymers Synthesized by a Sequential ROP-to-RAFT Procedure Inherently Temper Neuroinflammation

**DOI:** 10.1021/acs.biomac.2c01365

**Published:** 2023-02-09

**Authors:** Zulfiye Y. Turhan, Richard d’Arcy, Farah El Mohtadi, Lorena Infante Teixeira, Nora Francini, Mike Geven, Valentina Castagnola, Aws Alshamsan, Fabio Benfenati, Nicola Tirelli

**Affiliations:** † Laboratory for Polymers and Biomaterials, Fondazione Istituto Italiano di Tecnologia, 16163 Genova, Italy; ‡ Division of Pharmacy and Optometry, School of Health Sciences, University of Manchester, Oxford Road, Manchester M13 9PT, United Kingdom; § Center for Synaptic Neuroscience and Technology, Fondazione Istituto Italiano di Tecnologia, 16132 Genova, Italy; ∥ IRCCS Ospedale Policlinico San Martino, 16132 Genova, Italy; ⊥ Department of Pharmaceutics, College of Pharmacy, King Saud University, P.O. Box 2457, Riyadh 11451, Saudi Arabia; # Nanobiotechnology Unit, College of Pharmacy, King Saud University, P.O. Box 2457, Riyadh 11451, Saudi Arabia

## Abstract

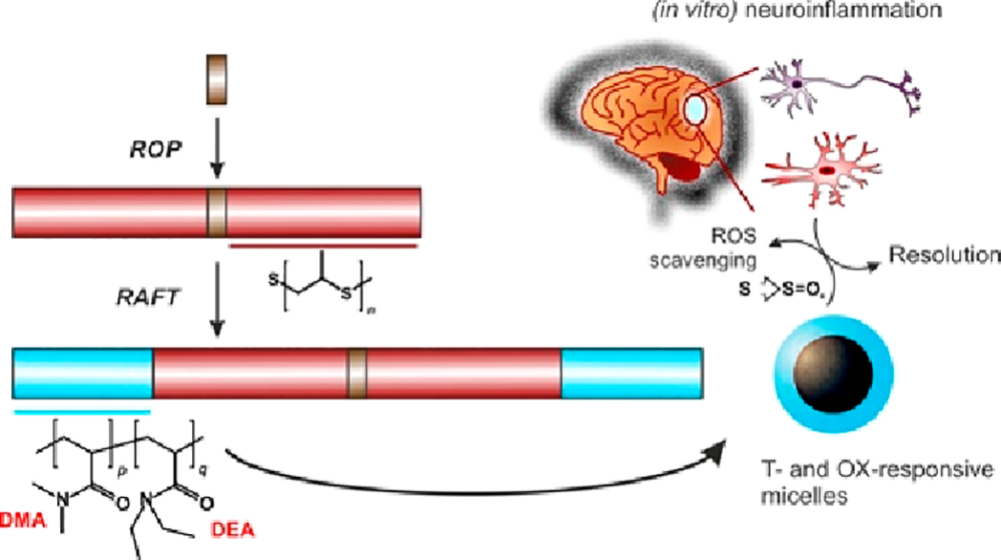

This study is about multiple responsiveness in biomedical materials. This typically implies “orthogonality” (i.e., one response does not affect the other) or synergy (i.e., one increases efficacy or selectivity of the other), but an antagonist effect between responses may also occur. Here, we describe a family of very well-defined amphiphilic and micelle-forming block copolymers, which show both oxidative and temperature responses. They are produced via successive anionic ring-opening polymerization of episulfides and RAFT polymerization of dialkylacrylamides and differ only in the ratio between inert (*N*,*N*-dimethylacrylamide, DMA) and temperature-sensitive (*N*,*N*-diethylacrylamide, DEA) units. By scavenging Reactive Oxygen Species (ROS), these polymers are anti-inflammatory; through temperature responsiveness, they can macroscopically aggregate, which may allow them to form depots upon injection. The localization of the anti-inflammatory action is an example of synergy. An extensive evaluation of toxicity and anti-inflammatory effects on *in vitro* models, including BV2 microglia, C8D30 astrocytes and primary neurons, shows a link between capacity of aggregation and detrimental effects on viability which, albeit mild, can hinder the anti-inflammatory potential (antagonist action). Although limited in breadth (e.g., only *in vitro* models and only DEA as a temperature-responsive unit), this study suggests that single-responsive controls should be used to allow for a precise assessment of the (synergic or antagonist) potential of double-responsive systems.

## Introduction

Inflammation is a natural response of the immune system to protect the body from damage potentially caused by pathogens or disrupted cellular processes. When controlled, it aims to restore homeostasis. When chronic, it is typically associated with a vast array of detrimental effects and an increased levels of oxidants;^1^ this oxidative stress is encountered in many pathologies, and just limiting this analysis to the central nervous system (CNS), it is encountered in Parkinson’s^2^ and Alzheimer’s diseases,^3^ multiple sclerosis,^4^ amyotrophic lateral sclerosis,^5^ spinal cord injury,^6^ and mental disorders.^7^ Anti-inflammatory approaches to the CNS are, however, hampered by a number of natural barriers, namely, the blood–brain barrier (BBB), blood–cerebrospinal fluid barrier (BCFB), and blood–brain–tumor barrier (BBTB); often, direct approaches (ideally requiring minimally invasive surgery followed by topical injection) are the only effective route, but their repeated use can be invasive and carries risks of infection. Therefore, the possibility of a localized depot formation, e.g., via a thermal gelation,^8^ is an appealing interventional avenue. For example, temperature-responsive systems composed of poly(ethylene glycol) (PEG)–poly(lactic-*co*-glycolic acid) (PLGA)^9^ and polymers of oligo- and di(ethylene glycol) methyl ether methacrylates^10^ were shown to be efficacious systems for *in situ,* body-temperature-activated formation of drug-loaded matrices in the treatment of spinal cord injury.

Herein, we explore the potential of temperature-dependent *in situ* depot formation in the context of polymers with inherent anti-inflammatory activity through Reactive Oxygen Species (ROS) scavenging.^11^ Our group has specifically studied organic polysulfides, in particular poly(propylene sulfide) (PPS), but also polysulfoxides;^12^ we refer the reader to a recent review for an updated account on these and other sulfur-based ROS scavenging polymers.^13^ The oxidation of hydrophobic thioethers (sulfides) by ROS, producing polar sulfoxides or sulfones, swells or solubilizes these nanomaterials, thereby allowing for an inflammatory-driven release of any encapsulated drug, which can synergize or add to the direct anti-inflammatory effects of ROS removal.^14^ Here, using *in vitro* models of CNS inflammation, we aimed to ascertain any synergistic or antagonistic outcomes of oxidant scavenging and temperature-induced aggregation.

Such double temperature- and oxidation-responsive macromolecules typically have amphiphilic structures. Here, we have also focused on the biocompatibility of their hydrophilic components. Poly(ethylene glycol) (PEG) is a gold standard in this area, due to its biocompatibility and “stealth” character; indeed, in previous studies we have produced PPS in a variety of morphologies (from cross-linked nanoparticles^15^ to self-assembled amphiphilic aggregates^14^) in a variety of multiblock combinations with PEG. However, in the last 15 years, it has become increasingly evident that anti-PEG antibodies naturally occur, are rather widespread, and lead to the accelerated blood clearance (ABC) of PEGylated formulations.^16,17^ In this study, as a PEG replacement, we have focused on poly(*N*,*N*-dialkylacrylamides), in particular poly(*N*,*N*-dimethylacrylamide) (PDMA) and poly(*N*,*N*-diethylacrylamide) (PDEA). For example, when decorating the surface of liposomes, PDMA has been shown not to induce ABC effect or IgM production upon repeated administration in rats^18^ (the same study showing a similar behavior also for other polymers including poly(2-hydroxypropyl methacrylamide) or poly(*N*-vinylpyrrolidone)).

Indeed, PDMA has been widely employed as an alternative to PEG.^19,20^ In particular, the group of Duvall has combined PDMA with PPS to yield micelle-forming block copolymers, where the hydrophilic segments are composed solely of PDMA^21^ or of PDMA/poly(*N*-isopropylacrylamide) (PNIPAm) diblocks.^22,23^ The latter composition combined the hydrophilicity and biocompatibility of PDMA with the inverse temperature gelation of PNIPAm, which may allow *in situ* gelation after injection and thereby the spatial resolution of the ROS scavenging activity of the system. For this very purpose, oxidation/temperature double responsive systems have been studied in recent years chiefly using PNIPAm^22,24^ or related systems.^25^ However, the literature in the field is scarce in providing comprehensive studies, lacking for example negative controls that depict no thermal gelation for comparison of such systems.

PDEA, on the one hand, is structurally analogous to PDMA (and the monomers should have a virtually identical polymerizability) but, on the other hand, is a thermally sensitive macromolecule,^26^ functionally similar to PNIPAm.^27^ It exhibits a lower critical solution temperature (LCST) in the region of 32–34 °C, slightly decreasing with molecular weights >10^4^ Da,^28^ and broadly independent of the macromolecular architecture (essentially constant in homopolymers,^29^ surface-immobilized gels,^30^ or block copolymeric nanogels^26^). In DEA/DMA random copolymers, DMA content increases the LCST.^31^ If this role is maintained also in PPS-containing structures, this parameter may provide a fine control over the range of thermal response.

**Scheme 1:**
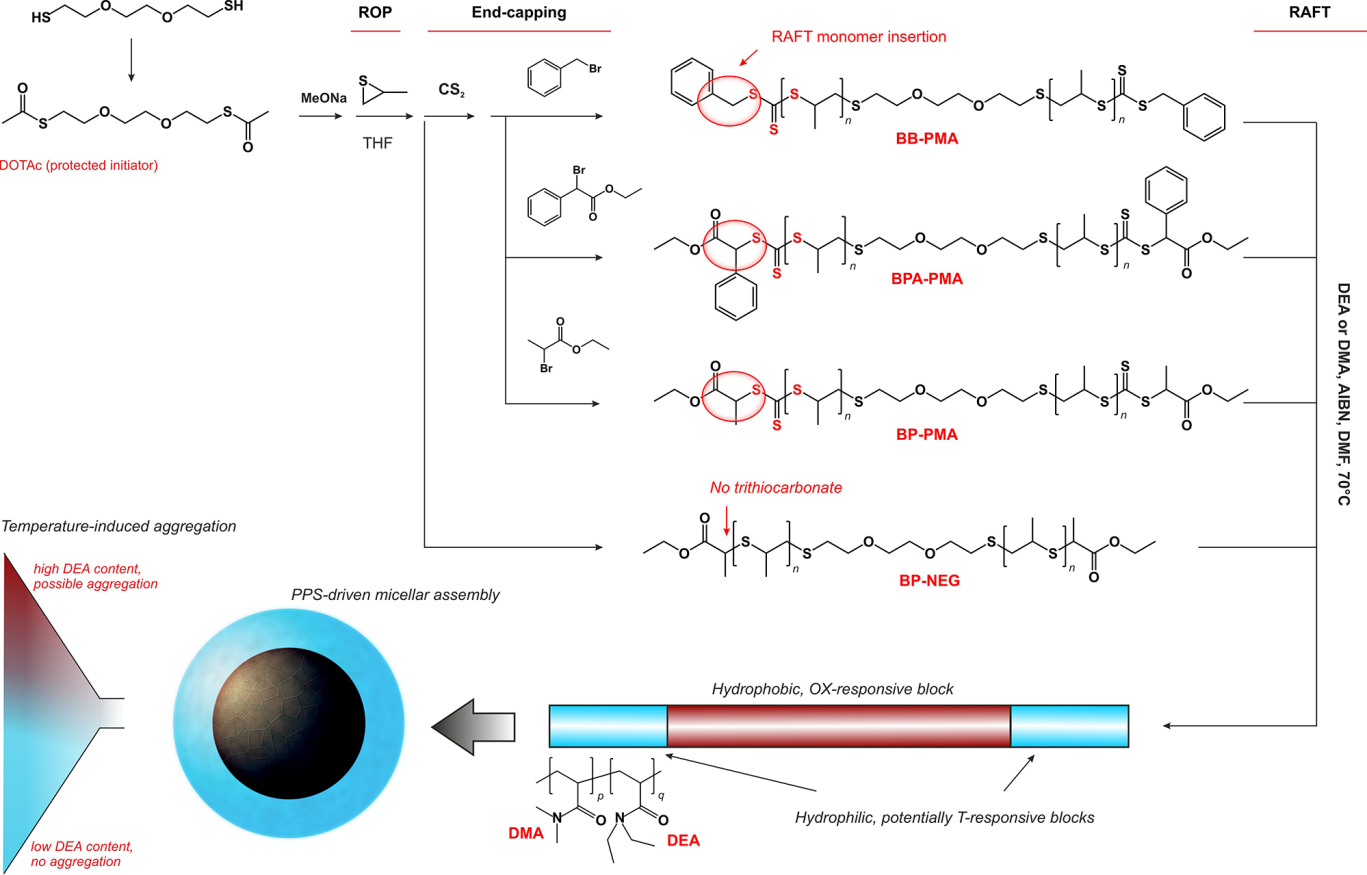
Synthetic Steps Leading to the Preparation of PPS-MacroRAFT agents (PMAs) and to Their Use for the Preparation of Thermally and Oxidation-Sensitive Amphiphilic Block Copolymers

Herein, we present a comprehensive structure–activity relationship, linking ROS scavenging activity and thermal gelation with DEA/DMA ratio for a family of precisely designed polymeric PPS-based amphiphiles. For their synthesis, we have sequentially combined two “living” polymerization mechanisms: the anionic ring-opening polymerization (ROP) of episulfides yields macromolecular chain transfer agents (macroRAFT agents) used in the reversible addition, fragmentation, and termination (RAFT) of the acrylamidic monomers (Scheme 1). By varying the DEA/DMA ratio, we aim to ascertain if the PDEA temperature-induced aggregation (potentially useful for *in situ* gelation) may present any adverse or synergic biological effects in the perspective of a CNS anti-inflammatory therapy. Such biomedical relevance was evaluated in terms of cell viability and inflammation biomarkers in 2D *in vitro* models with four different cell lines, namely macrophages, fibroblasts, astrocytes, and microglia, as well as with primary neurons, to achieve a comprehensive overview of this family of doubly responsive materials.

Of note, to identify the best performing PPS-MacroRAFT agent (PMA) structure, polymers with different substituents on the trithiocarbonate group were produced (a benzyl residue on BB-PMA, an aromatic β ester on BPA-PMA, and an aliphatic β ester on BP-PMA), in addition to a negative control without trithiocarbonate (BP-NEG). The overall aim was to produce well-defined macromolecules differing only in the DEA/DMA ratio and therefore in their potential for large-scale aggregation in water.

## Experimental Section

### Materials, (Bio)Chemicals, and Cells

All chemicals were used as received from suppliers unless otherwise stated. Dichloromethane (DCM), tetrahydrofuran (THF), *N*,*N*-dimethylformamide (DMF), tributylphosphine (TBP), and methanol were purchased from Thermo Fisher Scientific (Loughborough, UK). Sodium sulfate anhydrous (Na_2_SO_4_), acetic acid, basic alumina, molecular sieves 4 Å, and 30 wt % hydrogen peroxide solution (H_2_O_2_) were purchased from Merck (Darmstadt, Germany). Nile Red was purchased from FluoroChem (Hadfield, UK). All other chemicals and solvents were purchased from Sigma-Aldrich (Gillingham, UK). The inhibitor (monomethyl ether hydroquinone) was removed from *N*,*N*-dimethylacrylamide (DMA) and *N*,*N*-diethylacrylamide (DEA) by stirring with inhibitor removers (for removing hydroquinone and monomethyl ether hydroquinone) for an hour at room temperature followed by column filtration through basic alumina. The monomers DEA and DMA were stored at −20 °C prior to reactions. DMF was dried overnight on molecular sieves 4 Å. 2,2′-azobis(2-methylpropionitrile) (AIBN) was recrystallized from methanol. The rest of the chemicals were used without further purification.

Human dermal fibroblasts (neonatal, but usually defined as adult, HDFa), lipopolysaccharides (LPS) from *Escherichia coli* 02B:B6, Hank’s balanced salt solution (HBSS), neurobasal medium, GlutaMAX, Pierce BCA Protein Assay Kit, CyQUANT MTT cell viability assay, and TNF-α Mouse Uncoated ELISA Kit were purchased from Thermo Fisher Scientific (Leicestershire, UK); RAW264.7 macrophages, BV2+ microglia, and C8D30 astrocytes were purchased from ATCC. Dulbecco’s modified Eagle’s medium (DMEM), DMEM-high glucose (no phenol red), fetal bovine serum (FBS), l-glutamine 200 mM (×100), penicillin–streptomycin, and poly(l-lysine) (PLL) were purchased from Invitrogen (Paisley, UK). Cytotoxicity Detection Kit (LDH) was purchased from Roche (through Sigma-Aldrich (Milan, Italy)). The MTS proliferation assay (CellTiter 96 Aqueous One Solution Cell Proliferation Assay) was purchased from Promega (Madison, WI, USA). C57BL/6J mice were purchased from Charles River Laboratories Italia s.r.l. (Calco, Italy).

### Physico-chemical Characterization

#### ^1^H NMR Spectroscopy

^1^H NMR spectra were recorded on 15 mg/mL solutions in deuterated chloroform using a 400 MHz Bruker spectrometer (Bruker UK Limited, UK). ^13^C NMR spectra were recorded on 4-mg/mL solutions in deuterated chloroform using a 500 MHz Bruker spectrometer (Bruker UK Limited, UK).

#### FT-IR Spectroscopy

Spectra were recorded in ATR mode (Golden Gate) on a Tensor 27 Bruker spectrometer (Bruker UK Limited, UK) equipped with a 3000 Series TM High Stability Temperature Controller with RS232 Control (Specac, UK).

#### Mass Spectrometry

MALDI-ToF mass spectra were recorded on a Bruker UltrafleXtreme apparatus, operating in positive ionization mode at 20 kV and 80% laser output. PPS macroRAFT agents were dissolved in THF at a concentration of 10 mg/mL. The THF solutions contained also 4′-hydroxyazobenzene-2-carboxylic acid or dithranol at a 0.1 M concentration in the presence of 0.1 M of NaTFA or no salt. Appropriate volumes of sample and matrix solutions were mixed to obtain ratios of 1:1 or 1:2 (sample/matrix v/v). Appropriate volumes of NaTFA or THF (for no salt condition) were added to the sample and matrix mixture to obtain a 1:10 ratio (salt/sample&matrix v/v). An aliquot of 2 μL of each sample/matrix mixture was spotted onto a Bruker BigAnchor steel and slowly dried to allow matrix crystallization at ambient conditions. Average molecular weights *M̅*_n_ and *M̅*_w_ were directly calculated from the peak list of the mass spectra by the Bruker MS software PolyTools 2.0. The optimization of the analytical conditions is described in Supporting Information, section 1.5SI and Figures S5 to S13.

#### Gel Permeation Chromatography (GPC)

The analysis was performed using triple detection conditions with either a GPC50 (Polymer Laboratories, UK; THF mobile phase) or a Malvern OmniSec System (Malvern, UK; DMF mobile phase). Molecular weight and molecular weight distribution of PPS polymers were determined using a PLgel 5 μm MIXED-D column in series with a PLgel 3 μm MIXED-E operating online at 30 °C. THF was used as an eluent at a flow rate of 1.0 mL/min. Two linear polystyrene standards (Malvern, UK) for calibration (narrow) and for verification (broad) were used for the analysis of the polymers. Block copolymers of PPS with polyacrylamides were analyzed using a D3000 column operating online at 50 °C with HPLC grade DMF containing 0.1% LiBr as the mobile phase at a flow rate of 0.8 mL/min. Calibration was performed using a poly(methyl methacrylate) standard.

#### Shear Rheology

Dispersions (250 mg/mL) were prepared by mixing appropriate quantities of the polymers and deionized water at room temperature. The gel points of these dispersions were determined as the temperature where *G*′ (storage modulus) = *G*′′ (loss modulus) in experiments where the temperature was increased 2 °C/min in the interval 20–60 °C, using a HAAKE RheoStress 6000 (Thermo Scientific, Waltham, Massachusetts, USA) rheometer equipped with a Peltier temperature control unit. A cone–plate geometry with a diameter of 60 mm and a cone angle of 1° was used with a gap of 52 μm. All measurements were performed in the linear viscoelastic region (1 Hz, 0.1 Pa).

#### Dynamic Light Scattering (DLS)

Analysis was performed on micellar dispersions at a concentration of 1 mg/mL in deionized water using Zetasizer Nano ZS Instrument (Model ZEN3500, Malvern Instruments Ltd., UK) at an angle of 173° and a temperature of 25 °C.

#### Transmission Electron Microscopy (TEM)

Micellar dispersions (0.1 mg/mL) of the polymers in deionized water were dropcast onto ultrathin carbon/Formvar coated copper 150 mesh grids and stained with 1% uranyl acetate in water for 60 s before being analyzed with a JEOL JEM-1011 TEM, equipped with a W filament operating at 100 kV.

#### Determination of Critical Aggregation Concentration (CAC)

Block copolymer dispersions, 200 μL in deionized water (concentrations ranging from 0.0001 to 10 mg/mL), were mixed with 10 μL of a 3 × 10^–5^ M solution of Nile Red in THF in each well of a 96-well plate. The plate was protected from light, and THF was allowed to evaporate overnight. The Nile Red fluorescence was recorded on a Synergy2 Biotek plate reader with Gen5 software at 37 °C, using an excitation filter at 540 ± 25 nm and an emission filter at 620 ± 40 nm. The emission intensity at high and low concentration was fitted with linear equations, and their intersection was defined as the CAC (*n* = 3).

#### Determination of Cloud Point

Block copolymer dispersions in deionized water (200 μL) at concentrations of 1, 10, 50, 100, and 200 mg/mL in a 96-well plate were gradually heated from 24 to 45 °C. The optical densities at 500 nm were recorded on a Synergy2 Biotek plate reader with Gen5 software at every 1 °C increase in temperature with 2 min equilibration at each temperature before measurement (*n* = 3).

#### Oxidation Responsiveness

Nile Red-loaded polymer dispersions in deionized water were prepared and introduced in wells of a 96-well plate and mixed with hydrogen peroxide, obtaining a final volume of 200 μL, a thioether concentration of 5 mM (corresponding to a 0.4 mg/mL polymer concentration), and a hydrogen peroxide concentration of 40 mg/mL (4 wt %, 1.18 M). Controls without hydrogen peroxide were used to consider the possible decrease of Nile Red emission over time and were used to estimate the fraction of unbleached dye at all time points. The normalized fluorescence fraction was calculated as the [emission intensity at a given point] ÷ [emission intensity at time = 0 × the fraction of unbleached dye] (*n* = 3).

#### Vial Inversion Test

Block copolymer dispersions in deionized water (50, 100, 200, and 250 mg/mL) were prepared in small vials. The samples were heated in an incubator from 25 to 40 °C with equilibration time of 10 min.

### Preparative Operations

#### PPS-Based MacroRAFT Agents

All experiments were performed in a 12-position Radley’s carousel parallel reactor, purging it with argon for 5 min prior to the reactions. THF was degassed for 45 min with argon prior to the reactions. After each reaction, volatiles were removed *in vacuo*, and the product was dissolved in 30 mL DCM and extracted 3 times with 6 mL brine, dried over Na_2_SO_4_, filtered, and concentrated *in vacuo*. Each concentrate was then extracted 3 times with 5 mL of methanol. The resulting oil was dried in a Genevac EZ2 Elite centrifugal evaporator overnight at 40 °C. The final products were analyzed by ^1^H NMR, ^13^C NMR, FT-IR, GPC, and MALDI-ToF. Mass recovery (wt %) was calculated as the mass of polymer recovered divided by the theoretical mass of the polymer as determined using the composition measured by ^1^H NMR (based on the degree of polymerization and end-capping yields calculated) × 100.

##### BB-Capped PPS-MacroRAFT Agent (BB-PMA)

DOTAc (76.3 mg, 0.29 mmol) , TBP (0.215 mL, 0.86 mmol, 1.5 equiv per thioacetate), and 1.2 mL (0.6 mmol, 1.05 equiv per thioacetate) of a 0.5 M sodium methoxide solution in methanol were added in 10 mL of THF. The mixture was stirred for 15 min, then 850 mg (11.46 mmol, 20 equiv per thioacetate) of PS was introduced in the mixture, and the reagents were allowed to react for 45 min at room temperature. 0.14 mL (2.3 mmol, 4 equiv per thioacetate) of carbon disulfide was introduced and allowed to react for a further 15 min. Benzyl bromide (BB) (490 mg, 2.87 mmol, 5 equiv per thioacetate) was added and reacted overnight at room temperature; 722 mg (yield 66 wt %) of a yellow oily material was obtained.

^1^H NMR (CDCl_3_): δ = 1.35–1.45 (d, CH_3_ in PPS chain), 2.55–2.70 (m, PPS chain: 1 diastereotopic CH_2_), 2.71–2.78 (t, 4H, −SCH_2_CH_2_OCH_2_CH_2_OCH_2_CH_2_S−), 2.85–3.05 (m, PPS chain: CH and 1 diastereotopic CH_2_), 3.61–3.68 (m, 8H, −S–CH_2_–CH_2_–O–CH_2_–CH_2_–O–CH_2_–CH_2_–S−), 4.58–4.65 (s, 4H, −C(=S)–S–CH_2_–Ph), 7.30–7.38 ppm (m, 10H, phenyl protons).

^13^C NMR (CDCl_3_): δ = 20.8 (CH_3_ in PPS chain), 32.4 (−S–CH_2_–CH_2_–O–CH_2_–CH_2_–O–CH_2_–CH_2_–S−), 38.4 (CH_2_ in PPS chain), 41.2 (CH in PPS chain), 46.6 (CH_2_ in benzyl), 70.4 (−S–CH_2_–CH_2_–O–CH_2_–CH_2_–O–CH_2_–CH_2_–S−), 71.2 (−S–CH_2_–CH_2_–O–CH_2_–CH_2_–O–CH_2_–CH_2_–S−), 127.8 (phenyl carbon in *para* position), 128.7 (phenyl carbons in *ortho* position), 129.3 (phenyl carbons in *meta* position), 135 (phenyl carbon in *ipso* position), 222.6 ppm (−C(=S)–S–CH_2_–Ph).

FT-IR (ATR): ν̅ = 2958 (ν_as_ CH_3_), 2919 (ν_as_ CH_2_), 2865 (ν_s_ CH_3_ and ν_s_ CH_2_), 1370 (ν_as_ C–O–C), 820 (ν_as_ C=S), 734 cm^–1^ (ν_as_ C–S).

GPC (THF): *M̅*_n_ = 3100 g/mol, *Đ* = 1.17.

MALDI-ToF (HABA matrix with NaTFA at 1:2 polymer-to-matrix ratio in THF): *M̅*_n_ = 3700 g/mol, *Đ* = 1.01.

##### BPA-Capped PPS-MacroRAFT Agent (BPA-PMA)

DOTAc (76.3 mg, 0.29 mmol), TBP (0.215 mL, 0.86 mmol, 1.5 equiv per thioacetate), and 1.2 mL (0.6 mmol, 1.05 equiv per thioacetate) of a 0.5 M sodium methoxide solution in methanol were added in 5 mL of THF. The mixture was stirred for 15 min, then 850 mg (11.46 mmol, 20 equiv per thioacetate) of PS was introduced in the mixture, and the reagents were allowed to react for 45 min at room temperature. Carbon disulfide (0.14 mL, 2.3 mmol, 4 equiv per thioacetate) was introduced and allowed to react for a further 15 min. Ethyl α-bromophenylacetate (BPA; 697 mg, 2.87 mmol, 5 equiv per thioacetate) was added and reacted overnight at room temperature; 703 mg (yield 65 wt %) of a yellow oily material was obtained.

^1^H NMR (CDCl_3_): δ = 1.22–1.32 (t, 6H, CH_3_–CH_2_–O–C(=O)–CH(Ph)–S−), 1.35–1.45 (d, CH_3_ in PPS chain), 2.55–2.70 (m, PPS chain: 1 diastereotopic CH_2_), 2.71–2.78 (t, 4H, −SCH_2_CH_2_OCH_2_CH_2_OCH_2_CH_2_S−), 2.85–3.05 (m, PPS chain: CH and 1 diastereotopic CH_2_), 3.61–3.68 (m, 8H, −S–CH_2_–CH_2_–O–CH_2_–CH_2_–O–CH_2_–CH_2_–S−), 4.11–4.35 (m, 4H, CH_3_–CH_2_–O–C(=O)–CH(Ph)–S−), 5.77–5.81 (s, 2H, CH_3_–CH_2_–O–C(=O)–CH(Ph)–S−), 7.33–7.47 ppm (m, 10H, phenyl protons).

^13^C NMR (CDCl_3_): δ = 14.1 (CH_3_–CH_2_–O–C(=O)–CH(Ph)–S−), 20.8 (CH_3_ in PPS chain), 32.4 (−S–CH_2_–CH_2_–O–CH_2_–CH_2_–O–CH_2_–CH_2_–S−), 38.4 (CH_2_ in PPS chain), 41.2 (CH in PPS chain), 48.4 (CH_3_–CH_2_–O–C(=O)–CH(Ph)–S−), 61.9 (CH_3_–CH_2_–O–C(=O)–CH(Ph)–S−), 70.4 (−S–CH_2_–CH_2_–O–CH_2_–CH_2_–O–CH_2_–CH_2_–S−), 71.2 (−S–CH_2_–CH_2_–O–CH_2_–CH_2_–O–CH_2_–CH_2_–S−), 128.8 (phenyl carbon in *para* position), 128.9 (phenyl carbons in *ortho* position), 129 (phenyl carbons in *meta* position), 133 (phenyl carbon in *ipso* position), 168.7 (CH_3_–CH_2_–O–C(=O)–CH(Ph)–S−), 220.9 ppm (−CH(Ph)–S–C(=S)–S–CH_2_−).

FT-IR (ATR): ν̅ = 2958 (ν_as_ CH_3_), 2919 (ν_as_ CH_2_), 2865 (ν_s_ CH_3_ and ν_s_ CH_2_), 1736 (ν_as_ C=O), 1370 (ν_as_ C–O–C), 820 (ν_as_ C=S), 734 cm^–1^ (ν_as_ C–S).

GPC (THF): *M̅*_n_= 3811 g/mol, *Đ* = 1.09.

MALDI-ToF (dithranol matrix without cationizing agent at 1:1 polymer-to-matrix ratio in THF): *M̅*_n_ = 3600 g/mol, *Đ* = 1.01.

##### BP-Capped PPS-MacroRAFT Agent (BP-PMA)

DOTAc (76.3 mg, 0.29 mmol), TBP (0.43 mL, 1.72 mmol, 3 equiv per thioacetate), and 1.2 mL (0.6 mmol, 1.05 equiv per thioacetate) of a 0.5 M sodium methoxide solution in methanol were added in 5 mL of THF. The mixture was stirred for 15 min. Acetic acid (38 mg, 0.63 mmol, 1.1 equiv per thioacetate) and DBU (100 mg, 0.66 mmol, 1.15 equiv per thioacetate) were added followed by 850 mg (11.46 mmol, 20 equiv per thioacetate) of PS, and the reagents were allowed to react for 45 min at room temperature. Carbon disulfide (0.28 mL, 4.6 mmol, 8 equiv per thioacetate) was introduced and allowed to react for a further 15 min. Ethyl 2-bromopropionate (BP; 1038 mg, 5.7 mmol, 10 equiv per thioacetate) was added and reacted for 5 h at room temperature; 717 mg (yield: 70 wt %) of a yellow oily material was obtained.

^1^H NMR (CDCl_3_): δ = 1.27–1.32 (t, 6H, CH_3_–CH_2_–O–C(=O)–CH(CH_3_)–S−), 1.35–1.45 (d, CH_3_ in PPS chain and d, 6H, CH_3_–CH_2_–O–C(=O)–CH(CH_3_)–S−), 2.57–2.74 (m, PPS chain: 1 diastereotopic CH_2_), 2.74–2.79 (t, 4H, −SCH_2_CH_2_OCHCH_2_OCH_2_CH_2_S−), 2.85–3.01 (m, PPS chain: CH and 1 diastereotopic CH_2_), 3.61–3.68 (m, 8H, −S–CH_2_–CH_2_–O–CH_2_–CH_2_–O–CH_2_–CH_2_–S−), 4.18–4.32 (m, 4H, CH_3_–CH_2_–O–C(=O)–CH(CH_3_)–S−), 4.77–4.85 ppm (s, 2H, CH_3_–CH_2_–O–C(=O)–CH(CH_3_)–S−).

^13^C NMR (CDCl_3_): δ = 14.1 (CH_3_–CH_2_–O–C(=O)–CH(CH_3_)–S−), 20.8 (CH_3_ in PPS chain and CH_3_–CH_2_–O–C(=O)–CH(CH_3_)–S−), 32.4 (−S–CH_2_–CH_2_–O–CH_2_–CH_2_–O–CH_2_–CH_2_–S−), 38.4 (CH_2_ in PPS chain), 41.2 (CH in PPS chain), 48.4 (CH_3_–CH_2_–O–C(=O)–CH(CH_3_)–S−), 61.9 (CH_3_–CH_2_–O–C(=O)–CH(CH_3_)–S−), 70.4 (−S–CH_2_–CH_2_–O–CH_2_–CH_2_–O–CH_2_–CH_2_–S−), 71.2 (−S–CH_2_–CH_2_–O–CH_2_–CH_2_–O–CH_2_–CH_2_–S−), 168.7 (CH_3_–CH_2_–O–C(=O)–CH(CH_3_)–S−), 220.9 ppm (−CH(CH_3_)–S– C(=S)–S–CH_2_−).

FT-IR (ATR): ν̅ = 2958 (ν_as_ CH_3_), 2919 (ν_as_ CH_2_), 2865 (ν_s_ CH_3_ and ν_s_ CH_2_), 1736 (ν_as_ C=O), 1370 (ν_as_ C–O–C), 820 (ν_as_ C=S), 734 cm^–1^ (ν_as_ C–S).

GPC (THF): *M̅*_n_ = 3100 g/mol, *Đ* = 1.07.

MALDI-ToF (dithranol matrix without cationizing agent at 1:1 polymer-to-matrix ratio in THF): *M̅*_n_ = 2900 g/mol, *Đ* = 1.02.

##### BP-Capped PPS-MacroRAFT Agent without Trithiocarbonate (BP-NEG)

DOTAc (76.3 mg, 0.29 mmol), TBP (0.43 mL, 1.72 mmol, 3 equiv per thioacetate), and 1.2 mL (0.6 mmol, 1.05 equiv per thioacetate) of a 0.5 M sodium methoxide solution in methanol were added in 5 mL of THF. The mixture was stirred for 15 min. Acetic acid (38 mg, 0.63 mmol, 1.1 equiv per thioacetate) and DBU (100 mg, 0.66 mmol, 1.15 equiv per thioacetate) were added followed by 850 mg (11.46 mmol, 20 equiv per thioacetate) of PS, and the reagents were allowed to react for 45 min at room temperature. Ethyl 2-bromopropionate (BP; 1038 mg, 5.7 mmol, 10 equiv per thioacetate) was added and reacted overnight at room temperature; 583 mg (yield: 53 wt %) of a yellow oily material was obtained.

^1^H NMR (CDCl_3_): δ = 1.27–1.32 (t, 6H, CH_3_–CH_2_–O–C(=O)–CH(CH_3_)–S−), 1.35–1.45 (d, CH_3_ in PPS chain and d, 6H, CH_3_–CH_2_–O–C(=O)–CH(CH_3_)–S−), 2.57–2.74 (m, PPS chain: 1 diastereotopic CH_2_), 2.74–2.79 (t, 4H, −SCH_2_CH_2_OCH_2_CH_2_OCH_2_CH_2_S−), 2.85–3.01 (m, PPS chain: CH and 1 diastereotopic CH_2_), 3.46–3.57 (m, 2H, CH_3_–CH_2_–O–C(=O)–CH(CH_3_)–S−), 3.61–3.68 (m, 8H, −S–CH_2_–CH_2_–O–CH_2_–CH_2_–O–CH_2_–CH_2_–S−), 4.18–4.32 (m, 4H, CH_3_–CH_2_–O–C(=O)–CH(CH_3_)–S−), 4.77–4.85 ppm (s, 2H, CH_3_–CH_2_–O–C(=O)–CH(CH_3_)–S−).

^13^C NMR (CDCl_3_): δ = 14.1 (CH_3_–CH_2_–O–C(=O)–CH(CH_3_)–S−), 20.8 (CH_3_ in PPS chain and CH_3_–CH_2_–O–C(=O)–CH(CH_3_)–S−), 32.4 (−S–CH_2_–CH_2_–O–CH_2_–CH_2_–O–CH_2_–CH_2_–S−), 38.4 (CH_2_ in PPS chain), 41.2 (CH in PPS chain), 48.4 (CH_3_–CH_2_–O–C(=O)–CH(CH_3_)–S−), 61.9 (CH_3_–CH_2_–O–C(=O)–CH(CH_3_)–S−), 70.4 (−S–CH_2_–CH_2_–O–CH_2_–CH_2_–O–CH_2_–CH_2_–S−), 71.2 (−S–CH_2_–CH_2_–O–CH_2_–CH_2_–O–CH_2_–CH_2_–S−), 168.7 (CH_3_–CH_2_–O–C(=O)–CH(CH_3_)–S−).

FT-IR (ATR): ν̅ = 2958 (ν_as_ CH_3_), 2919 (ν_as_ CH_2_), 2865 (ν_s_ CH_3_ and ν_s_ CH_2_), 1736 (ν_as_ C=O), 1370 (ν_as_ C–O–C), 734 cm^–1^ (ν_as_ C–S).

GPC (THF): *M̅*_n_ = 3000 g/mol, *Đ* = 1.08.

MALDI-ToF (dithranol matrix without cationizing agent at 1:1 polymer-to-matrix ratio in THF): *M̅*_n_ = 3100 g/mol, *Đ* = 1.01.

#### Synthesis of PPS-DMA/DEA Block Copolymers

All experiments were performed in a 12-position Carousel parallel reactor. In a typical polymerization, PPS-macroRAFT agent (PMA), AIBN, monomer, and 5 mg/mL of trioxane as an internal standard were dissolved in dry DMF. The mixture was degassed with argon for 45 min and reacted at 70 °C under argon atmosphere. Samples were collected during the reactions for conversion analysis via ^1^H NMR on nonpurified samples by normalizing to the trioxane at 5.1 ppm and measuring the consumption of monomer via the loss of the peak at 5.6 ppm. The polymerizations were stopped by exposure to air and placing on ice when monomer consumption reached a plateau. The reaction solution was diluted in deionized water followed by dialysis and freeze-drying. The purified block copolymers were characterized by ^1^H NMR and GPC.

##### DMA Homopolymerization with BB-PMA

BB-PMA (200 mg, 0.058 mmol), AIBN (0.9 mg, 0.0058 mmol, 0.1 equiv per BB-PMA), DMA (570 mg, 5.76 mmol, 100 equiv per BB-PMA), and 5 mg/mL of trioxane as an internal standard were dissolved in 6 mL of dry DMF and reacted for 8 h; 648 mg (yield: 93 wt %) of white fluffy material was obtained.

^1^H NMR (CDCl_3_): δ = 1.05–1.27 (CH_2_ in PDMA chain, *m* diads), 1.27–1.37 (d, CH_3_ in PPS chain), 1.38–1.64 (CH_2_ in PDMA chain, *r* diads), 1.64–1.91 (CH_2_ in PDMA chain, *m* diads), 2.15–2.72 (m, Ph–CH_2_–[CH_2_–CH(C(=O)–N(CH_3_)(CH_3_))]_*n*_–S–C(=S)–; CH in PDMA chain; t, SCH_2_CH_2_OCH_2_CH_2_OCH_2_CH_2_S–; and 1 diastereotopic CH_2_ in PPS chain), 2.71–3.16 (CH_3_ in PDMA chain; m, CH in PPS chain; and 1 diastereotopic CH_2_ in PPS chain), 3.53–3.63 (m,–S–CH_2_–CH_2_–O–CH_2_–CH_2_–O–CH_2_–CH_2_–S−), 7.03–7.15 (m, phenyl protons in *para* and *ortho* positions), 7.15–7.22 ppm (m, phenyl protons in *meta* position).

GPC (DMF): *M̅*_n_ = 10600 g/mol, *Đ* = 1.15.

##### DMA Homopolymerization with BPA-PMA

BPA-PMA (200 mg 0.054 mmol), AIBN (0.9 mg, 0.0054 mmol, 0.1 equiv per BPA-PMA), DMA (537 mg, 5.4 mmol, 100 equiv per BPA-PMA), and 5 mg/mL of trioxane as an internal standard were dissolved in 6 mL of dry DMF and reacted for 78 h; 507 mg (yield: 80 wt %) of white fluffy material was obtained.

^1^H NMR (CDCl_3_): δ = 1.05–1.27 (CH_2_ in PDMA chain, *m* diads; t, CH_3_–CH_2_–O–C(=O)–CH(Ph)−), 1.27–1.37 (d, CH_3_ in PPS chain), 1.38–1.64 (CH_2_ in PDMA chain, *r* diads), 1.64–1.91 (CH_2_ in PDMA chain, *m* diads), 2.49–2.72 (t, SCH_2_CH_2_OCH_2_CH_2_OCH_2_CH_2_S–; 1 diastereotopic CH_2_ in PPS chain; and CH in PDMA chain), 2.71–3.16 (CH_3_ in PDMA chain; m, CH in PPS chain; and 1 diastereotopic CH_2_ in PPS chain), 3.53–3.63 (m,–S–CH_2_–CH_2_–O–CH_2_–CH_2_–O–CH_2_–CH_2_–S–; and t, CH_3_–CH_2_–O–C(=O)–CH(Ph)−), 3.91–4.10 ppm (q, CH_3_–CH_2_–O–C(=O)–CH(Ph)−).

GPC (DMF): *M̅*_n_ = 11800 g/mol, *Đ* = 1.08.

##### DMA Homopolymerization with BP-PMA

BP-PMA (200 mg, 0.061 mmol), AIBN (1 mg, 0.0061 mmol, 0.1 equiv per BP-PMA), DMA (606 mg, 6.1 mmol, 100 equiv per BP-PMA), and 5 mg/mL of trioxane as an internal standard were dissolved in 6 mL of dry DMF and reacted for 5 h; 751 mg (yield: 92 wt %) of white fluffy material was obtained.

^1^H NMR (CDCl_3_): δ = 1.05–1.27 (CH_2_ in PDMA chain, *m* diads; t, CH_3_–CH_2_–O–C(=O)–CH(CH_3_)−); and t, CH_3_–CH_2_–O–C(=O)–CH(CH_3_)−), 1.27–1.37 (d, CH_3_ in PPS chain), 1.38–1.64 (CH_2_ in PDMA chain, *r* diads), 1.64–1.91 (CH_2_ in PDMA chain, *m* diads), 2.21–2.72 (t, SCH_2_CH_2_OCH_2_CH_2_OCH_2_CH_2_S–; 1 diastereotopic CH_2_ in PPS chain; m, CH_3_–CH_2_–O–C(=O)–CH(CH_3_)–S−); and CH in PDMA chain), 2.71–3.16 (CH_3_ in PDMA chain; m, CH in PPS chain; and 1 diastereotopic CH_2_ in PPS chain), 3.53–3.63 (m,–S–CH_2_–CH_2_–O–CH_2_–CH_2_–O–CH_2_–CH_2_–S−), 3.91–4.10 ppm (q, CH_3_–CH_2_–O–C(=O)–CH(CH_3_)−).

GPC (DMF): *M̅*_n_ = 13700 g/mol, *Đ* = 1.18.

##### DEA/DMA Copolymerizations (BP-PMA)

BP-PMA (200 mg, 0.058 mmol), AIBN (1 mg, 0.0058 mmol, 0.1 equiv per BP-PMA), 5 mg/mL of trioxane as an internal standard, and monomers were dissolved in 6 mL of dry DMF and reacted for 5 h. Monomer quantities and yields: (A) 100% DEA, 744 mg DEA (5.8 mmol, 100 equiv per BP-PMA); yield 869 mg (79 wt %). (B) 75:25 DEA/DMA, 145 mg (1.46 mmol, 25 equiv per BP-PMA) of DMA, 558 mg (4.39 mmol, 75 equiv per BP-PMA) of DEA; yield 840 mg (92 wt %). (C) 50:50 DEA/DMA, 290 mg (2.92 mmol, 50 equiv per BP-PMA) of DMA, 372 mg (2.92 mmol, 50 equiv per BP-PMA) of DEA; yield 776 mg (90 wt %). (D) 25:75 DEA/DMA, 435 mg (4.39 mmol, 75 equiv per BP-PMA) of DMA, 186 mg (1.46 mmol, 25 equiv per BP-PMA) of DEA; yield 752 mg (97 wt %).

^1^H NMR (CDCl_3_): δ = 0.87–1.27 (t, 6H, CH_3_–CH_2_–O–C(=O)–CH(CH_3_)–; d, 6H, CH_3_–CH_2_–O–C(=O)–CH(CH_3_)–; CH_3_ in PDEA chain; and CH_2_ in PDEA and PDMA chains, *m* diads), 1.27–1.37 (d, CH_3_ in PPS chain), 1.38–1.64 (CH_2_ in PDEA and PDMA chains, *r* diads), 1.64–1.91 (CH_2_ in PDEA and PDMA chains, *m* diads), 2.21–2.72 (t, 4H, SCH_2_CH_2_OCH_2_CH_2_OCH_2_CH_2_S–; 1 diastereotopic CH_2_ in PPS chain; m, CH_3_–CH_2_–O–C(=O)–CH(CH_3_)–S–; DEA and DMA chain CH), 2.72–3.54 (m, CH in PPS chain; 1 diastereotopic CH_2_ in PPS chain; DEA N–CH_2_–CH_3_; DMA N–CH_3_), 3.54–3.63 (m, – S–CH_2_–CH_2_–O–CH_2_–CH_2_–O–CH_2_–CH_2_–S−), 3.91–4.10 ppm (q, CH_3_–CH_2_–O–C(=O)–CH(CH_3_)−).

GPC (DMF): please refer to Table 2.

### Cell Cultures

RAW 264.7 macrophages and fibroblasts were cultured in DMEM supplemented with 10% FBS, 100 U/mL penicillin, 100 μg/mL streptomycin, 0.25 μg/mL amphotericin B, and 0.2 M l-glutamine at 37 °C in a humidified atmosphere of 5% CO_2_. BV2 microglia and C8D30 astrocytes were cultured in DMEM-high glucose (4500 mg/L glucose) supplemented with 10% FBS, 100 U/mL penicillin, 100 μg/mL streptomycin, 1 mM sodium pyruvate, and 2 mM l-glutamine at 37 °C in a humidified atmosphere of 5% CO_2_. Cell culture maintenance was performed by washing the cells with Dulbecco’s phosphate-buffered saline (PBS), followed by cell detachment using trypsin. The detached cells were then suspended in culture medium, counted using trypan blue dye, plated in culture plates, and cultured at 37 °C.

Primary cultures of mouse cortical neurons were prepared from embryos on day 17–18 of pregnancy, and all *in vivo* experiments were performed in accordance with the guidelines and regulations of the European Directive 2010/63/EU and approved by the Italian Ministry of Health (authorization code: 22418.N.KOL). Pregnant mice (C57BL/6J) were sacrificed, and the uterus was dissected out and placed in a sterile Petri dish with HBSS. Embryonic sacs were removed from uterus and transferred to a 6 cm HBSS-filled dish. Using a dissecting microscope, the brains of the embryos and then the cortices of those embryos were dissected out and were placed in a dish containing HBSS. Cortices were cut into small pieces and washed 3 times with HBSS. Ten milliliters of 0.25% trypsin was added and incubated for 25–30 min in a water bath at 37 °C. The solution was discarded, and the cortices were washed twice with HBSS followed by addition of Neurobasal medium supplemented with 5% FBS. The merged cell suspensions were filtered through a 40 μm filter. The cell culture plates were coated with 0.1 mg/mL poly(l-lysine) (PLL) overnight, washed with deionized water the day of the dissection, and dried. Cells were cultured in Neurobasal medium supplemented with 2% B-27, 100 U/mL penicillin, 100 μg/mL streptomycin, and l-glutamine at 37 °C in a humidified atmosphere of 5% CO_2_.

### Cell Viability

#### Cell Lines

For metabolic activity assay, the cells were seeded in 96-well plates (10^4^ cells per well for fibroblasts and macrophages, 2 × 10^4^ cells per well for BV2, and 3 × 10^4^ cells per well for C8D30) and allowed to adhere overnight. Cells were washed with PBS, and 200 μL of fresh medium with polymers at a concentration of 0.01, 0.05, 0.1, 0.5, 1, 5, and 10 mg/mL was added. After 24 h incubation, the medium was discarded, cells were gently washed with PBS and incubated for 1 h at 37 °C with CellTiter 96 Aqueous One Solution Reagent in medium prepared by following the manufacturer instructions, and the absorbance was measured at 490 nm using a plate reader. The solution was then removed, and cells were washed with PBS. To lyse the cells, 100 μL of RIPA buffer was added; the cells were kept −80 °C overnight followed by incubation at 37 °C for 1 h. The total protein content was determined using Pierce BCA Protein Assay Kit following the manufacturer instructions as follows: 25 μL of lysed cell solution was mixed with 200 μL of BCA working reagent, incubated at 37 °C for 30 min, and cooled to room temperature, and the absorbance was measured at 562 nm using a plate reader. The absorbance readings of the treated cells obtained from CellTiter 96 Aqueous One Solution Assay and Pierce BCA Protein Assay were normalized to the absorbance reading of the untreated cells. The ratio between the metabolic activity (%) and the protein content (%) was determined, and the results are expressed as the percentage of treated vs untreated cells.

#### Primary Neurons

Metabolic activity was assessed via a generic metabolic activity assay (MTT), and via a live–dead assay.

##### MTT assay

Primary cortical neurons were seeded at a density of 2 × 10^4^ cells per PLL-coated well (see above) of a 96-well plate, keeping them in culture (200 μL of medium) for 6 days. Then 100 μL of medium was removed, and 100 μL of polymer dispersions in medium at concentrations of 0.02, 0.1, 0.1, 1, and 2 mg/mL was added, to obtain final polymer concentrations of 0.01, 0.05, 0.1, 0.5, and 1 mg/mL, respectively. Cells were finally incubated for 24 and 48 h, and CyQUANT MTT Cell Viability Assay was performed following the manufacturer instructions with appropriate adaptations as follows: 100 μL of the medium was discarded, 10 μL of MTT stock solution was added, and the cells were incubated at 37 °C for 4 h followed by addition of 100 μL of SDS-HCl solution and further incubation at 37 °C for 4 h. The absorbance was measured at 570 nm. The results are expressed as the percentage of treated vs untreated cells.

##### Live/Dead Assay

Neurons were seeded at a density of 3 × 10^4^ cells per well on glass coverslips (12 mm diameter) that were precoated with 0.1 mg/mL of PLL (100 μL/well) on a 24-well plate (1 mL of medium) and kept in culture for 6 days. Then 500 μL of medium was removed and 500 μL of polymer dispersions in the medium at concentrations of 0.02, 0.1, 0.1, 1, and 2 mg/mL was added, to obtain final polymer concentrations of 0.01, 0.05, 0.1, 0.5, and 1 mg/mL, respectively. After 24 h incubation, 1 μL of propidium iodide, calcein-AM, and Hoechst dye solutions was added. All nuclei (DAPI channel, excitation at 345 nm and emission at 455 nm), live cells (FITC channel, excitation at 495 nm and emission at 518 nm), and dead cells (TRITC channel, excitation at 536 nm and emission at 617 nm) were visualized under a NIKON Eclipse T_i_ epifluorescence microscope using NIS-Elements imaging software and ImageJ for the image analysis; 3–5 random fields were imaged per culture well. The average metabolic activity per cell was calculated normalizing the MTT data against the number of attached (DAPI-positive) cells.

### Evaluation of Anti-inflammatory Effects

#### BV2 Microglia

Cells were seeded on 96-well plates at a density of 2 × 10^4^ cells per well and allowed to adhere overnight. The following day, cells were treated for 24 h with 0.5 μg/mL LPS in fresh DMEM before an additional 24 h incubation with DMEM containing 0.5 μg/mL LPS and 0.5 and 1 mg/mL polymers. Control groups consisted of untreated cells and LPS-stimulated cells with no polymer treatment. Culture supernatants were collected and centrifuged at 1000 rpm for 5 min and analyzed for the presence of TNF-α by ELISA, as well as for the measurement of LDH levels. BCA assay was performed to quantify the total protein content per well.

#### C8D30 Astrocytes

These cells were activated using conditioned medium (MCM) from LPS-stimulated BV2 cells. BV2 were seeded at a density of 1.5 × 10^5^ cells per well on a 24-well plate and allowed to adhere overnight. The following day, BV2 cells were treated for 24 h with 0.5 μg/mL LPS in fresh DMEM. The same day C8D30 astrocytes were seeded at a density of 3 × 10^4^ cells per well in a 96-well plate and allowed to adhere overnight. On the next day, the supernatants of BV2 cells were collected and centrifuged at 1000 rpm for 5 min to remove the dead cells and debris. MCM was prepared by mixing 80% (v/v) of the supernatant collected after centrifugation with 20% (v/v) of fresh DMEM. The culture medium of C8D30 cells was replaced with 200 μL per well of MCM, and cells were incubated 24 h before an additional 24 h incubation with 0.5 or 1 mg/mL polymers in fresh MCM. Control groups consisted of untreated cells and MCM-stimulated cells with no polymer treatment. Supernatants were collected and analyzed as described above.

#### Primary Cortical Neurons

Primary neurons were activated using conditioned medium (MCM) from LPS-stimulated BV2 cells obtained as described above. Primary neurons were seeded at a density of 2 × 10^4^ cells per well on a 96-well plate (precoated with 0.1 mg/mL of PLL (200 μL/well), keeping them in culture (200 μL of medium) for 6 days. Then 100 μL of medium was removed, 100 μL of MCM per well was added, and cells were incubated for 24 h before an additional 24 h incubation with polymers (0.5 and 1 mg/mL) in fresh MCM. Control groups consisted of untreated cells and MCM stimulated cells with no polymer treatment. Supernatants were collected and analyzed as described above.

#### Enzyme-Linked Immunosorbent Assay (ELISA)

TNF-α levels were quantified by ELISA using mouse kits following the manufacturer’s instructions. Values were normalized against the total protein content per well, setting a 100% value for the level of membrane damage obtained by treating cells with MCM alone.

#### Lactate Dehydrogenase (LDH) Assay

Membrane damage after treatments was analyzed using an LDH assay kit according to the manufacturer’s instructions. In brief, supernatants were collected, LDH was measured, and optical densities were normalized against the total protein content per well, setting a 100% value for the level of membrane damage obtained by treating cells with MCM alone.

### Statistical Analysis

Data are presented as mean values ± standard error of the mean (SEM). Normality was assessed with the Shapiro–Wilk test. The statistical analysis employed one-way analysis of variance (ANOVA) test with Dunnett’s post hoc test or the Kruskal–Wallis test with Dunn’s post hoc test. Accepted levels of significance were *p < 0.05, **p < 0.01, ***p < 0.001, ****p < 0.0001. Statistical analyses were conducted using GraphPad Prism 9.3.1

## Results and Discussion

### Preparation of MacroRAFT Agents

Here, we have employed a protected dithiol initiator (DOTAc; for synthesis, see Supporting Information, section 1.1SI and Figure S1), whose thioacetates were first converted into active sodium thiolates via reaction with sodium methoxide. This procedure, together with the use of an *in situ* reducing agent such as tributylphosphine (TBP), aims to minimize the presence of disulfides in the polymerization environment, which would act as transfer agents in the episulfide ROP, causing the formation of polydisperse and/or wrongly end-capped polymers.^32,33^ The episulfide used as a monomer in this study (propylene sulfide, PS) was also synthesized, using a scaled-up and automatized procedure (see Supporting Information, section 1.2SI and Figure S2).

Upon addition of PS to the deprotected thiolate initiator, polymerization readily ensued; after 45 min CS_2_ was added, followed by end-capping using six different bromides for the one-pot in situ formation of a polysulfide macroRAFT agent. In detail, we employed a primary (benzyl bromide, BB), four secondary (ethyl α-bromophenylacetate, BPA; ethyl 2-bromopropionate, BP; 2-bromopropionamide, BPAM; 2-bromopropionitrile, BPN), and a tertiary bromide (ethyl α-bromoisobutyrate, BIB). From consensus guidelines,^34^ it is expected that the transfer coefficients of these groups will decrease in the order BPA > BIB ≫ BPN > BP ≈ BPAM > BB ≳ polysulfide chain. Indeed, a secondary radical-terminated PPS is less stable than any of the radicals (conjugated or more sterically hindered) possibly produced by this set of end-cappers.

**Figure 1:**
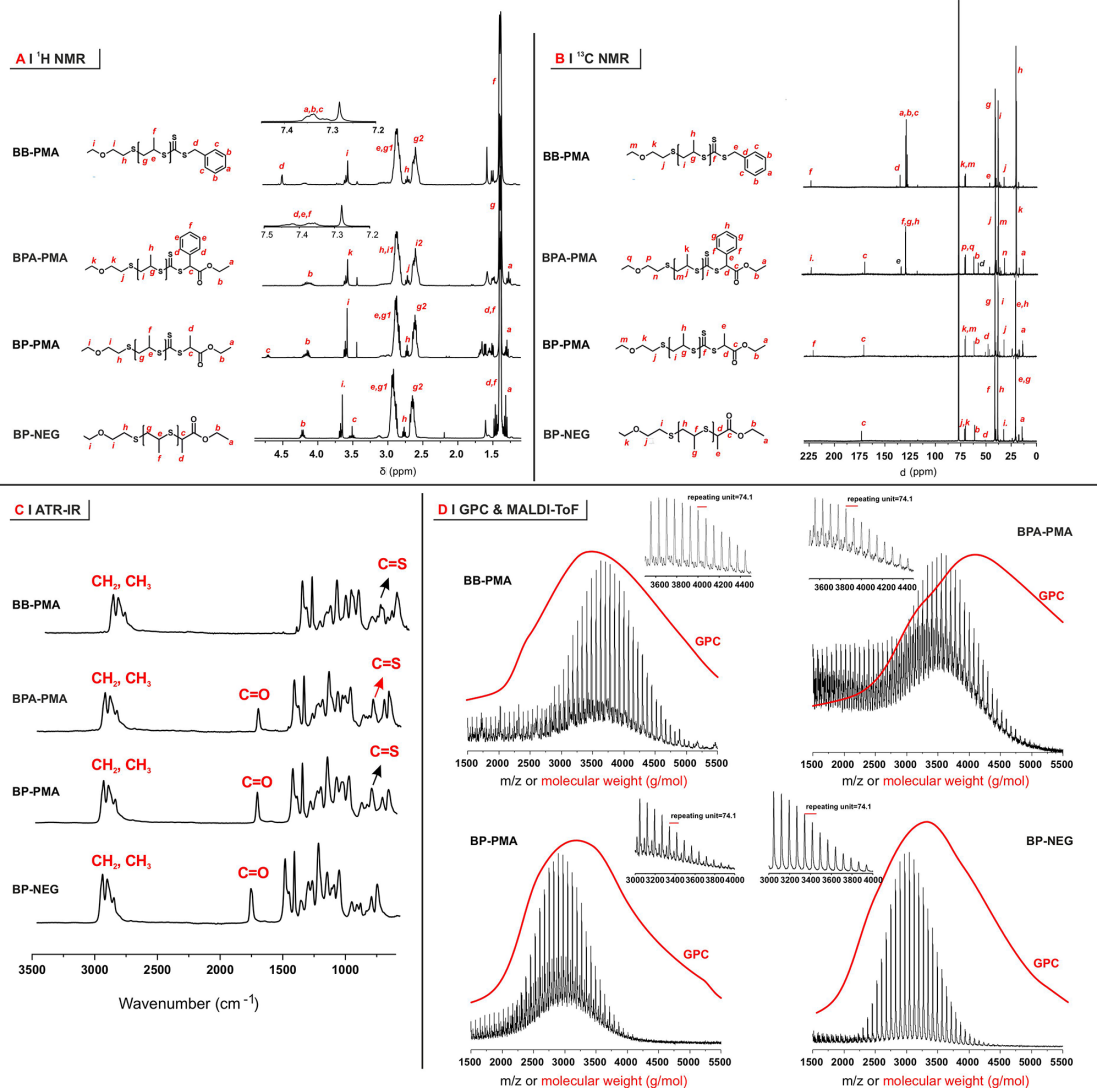
(A) ^1^H NMR spectra of PPS-macroRAFT agents (PMAs); the assignments are reported in red in the polymer structures on the left. (B) ^13^C NMR spectra of PMAs. (C) IR spectra of PMAs, with the most characteristic peaks highlighted. (D) Molecular weight distributions for the four PMAs obtained through GPC (red lines) and MALDI-ToF (black) in the 1500–5000 Da range. In the insets, the expanded views show spacing corresponding to the weight of PS units.

We have screened several reaction conditions to produce these trithiocarbonate derivatives (see Supporting Information, section 1.3SI and Table S1), with the aim to obtain quantitative end-capping and avoid multimodal molecular weight distribution, which may arise from uncapped PPS thiolates. As a result of this phase, well-defined poly(propylene sulfide) macroRAFT agents (PMAs) could only be produced using BB, BPA, and BP (BB-PMA, BPA-PMA, and BP-PMA (Figure 1, Table 1, and in section 1.4SI, Figure S3, in the Supporting Information).

**Table 1:** Physico-chemical Characterization of PPS-Based MacroRAFT Agents (PMAs)

	theoretical		^1^H NMR		GPC^d^		MALDI-ToF^e^	
polymer	DP	*M̅*_n_ (Da)^a^	*M̅*_n_ (Da)^b^	end-capping (%)^c^	*Đ*	*M̅*_n_ (Da)	*Đ*	*M̅*_n_ (Da)
BB-PMA	40	3500	3500	100	1.17	3100	1.01	3700
BPA-PMA	40	3700	3800	100	1.09	3800	1.01	3600
BP-PMA	40	3500	3400	100	1.07	3100	1.02	2900
BP-NEG	40	3300	3300	100	1.08	3000	1.01	3100

a Calculated assuming a 100% end-capping yield.

b Calculated from the CH_2_ resonance at 2.71 ppm, after normalization against the initiator resonance at 3.70 ppm.

c Calculated from the resonance of the different end-capper CH_2_ groups (4.64 ppm for BB-PMA, 4.31 ppm for BP-PMA, 4.32 ppm for BPA-PMA) (i.e., calculated from the fraction of the actual and theoretical integrals of that end-capping peak).

d Measured through triple-detection GPC analysis in THF.

e Dithranol was employed as matrix for BB-PMA, BP-PMA, and BP-NEG, with a polymer-to-matrix ratio of 1:1 (v/v) in the absence of a cationizing agent; HABA was employed as the matrix for BB-PMA, with a polymer-to-matrix ratio 1:2 (v/v) in the presence of a NaTFA; all spectra were in positive ion linear mode. For a complete overview of the optimization of MALDI-ToF conditions, please refer to Supporting Information, section 1.4SI.

A negative control with a terminal ethyl 2-propionate and no trithiocarbonate (BP-NEG) was produced under the same reaction conditions used for the synthesis of BP-PMA, minus the use of CS_2_. Of note, the MW distributions obtained through GPC and MALDI-ToF show minor discrepancies only for BPA-PMA, while for all other PMAs they have a remarkable agreement (Figure 1D).

Qualitatively, the incorporation of trithiocarbonates in the PMAs was confirmed through the presence of a resonance at 220.9 ppm in ^13^C NMR and an IR absorption at 820 cm^–1^ attributed to C=S groups. The near UV absorption of trithiocarbonates at 310 nm in principle can provide more quantitative information, but it is critical to choose a model compound with substituents similar to the target group. For example, the fully aliphatic bis(carboxymethyl)trithiocarbonate) is structurally similar to the trithiocarbonate in BP-PMA, and using the extinction coefficient of the former, the latter indeed appears to bear two C=S groups per chain, i.e., quantitative end-capping (see Supporting Information, section 1.4SI and Figure S4). BB-PMA and BPA-PMA, on the contrary, feature phenyl rings close to the trithiocarbonate group, which is likely different in extinction coefficient. However, since the reaction between PPS terminal thiols and CS_2_ was performed under identical conditions for all PMAs, we assumed them to be all quantitatively end-capped.

### Use of PMAs in RAFT Polymerization

**Figure 2:**
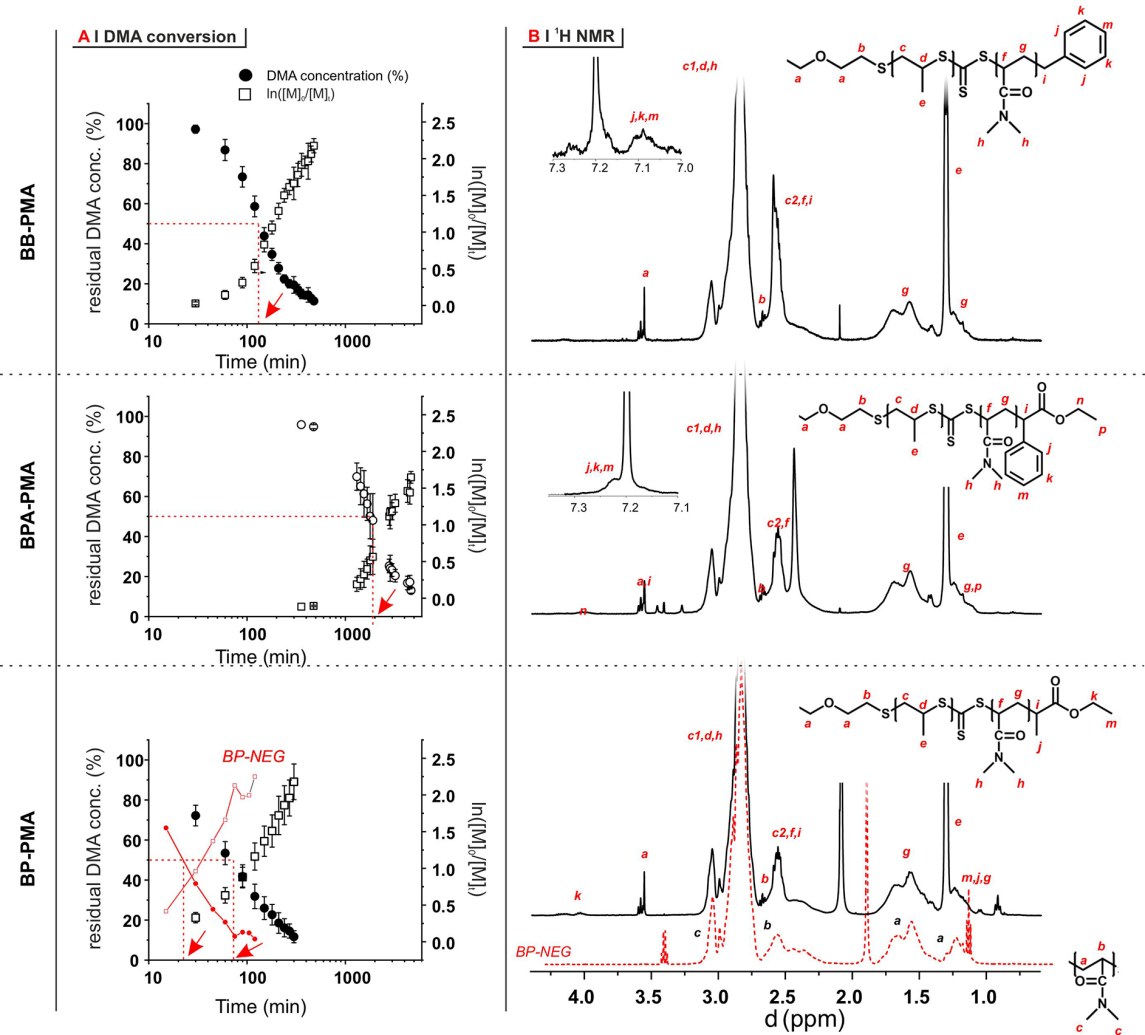
(A) DMA concentration (left axes) and pseudo-first-order kinetics graphs (right axes) for RAFT polymerization of DMA in the presence of BB-PMA (top), BPA-PMA (middle), and BP-PMA (bottom) (*n* = 3). The data for BP-NEG are reported for comparison in the BP-PMA graph, shown in red. For a rapid appreciation of the kinetics of the process, dashed red lines highlight the time point associated with 50% monomer consumption. (B) ^1^H NMR spectra for the products of polymerization experiments conducted for 8 h for BB-PMA, 78 h for BPA-PMA, 5 h for BP-PMA, and 2 h for BP-NEG.

The RAFT homopolymerization of DMA (AIBN, 70 °C) was employed to screen the three PMAs described above. In all experiments, DMA conversion was detected by ^1^H NMR as a function of time. With BB-PMA (Figure 2A, top) we observed an initial “inhibition” period, with no significant monomer consumption for almost 1 h; DMA polymerization then started, and the monomer was >80% converted in the following 5 h. Polymerization from BPA-PMA (Figure 2A, middle) had a much longer “inhibition” period (around 8 h) and was then much slower, requiring about 72 h to reach 80% monomer consumption. Only BP-PMA showed a classical pseudo-first-order kinetics, with prompt initiation and 88% DMA conversion within the first 5 h (Figure 2A, bottom). Nevertheless, despite differences in inhibition periods and kinetics of polymerization, each macroRAFT agent produced polymers of targeted molecular mass and low dispersity (*Đ* = 1.1–1.2), as observed in Table 2 by the agreement between the values obtained with GPC and the ones calculated by the conversion of functional groups detected by ^1^H NMR spectroscopy. Finally, polymerization in the presence of BP-NEG, on the other hand, led to a more rapid monomer consumption (red curve, Figure 2A, bottom left), providing a polymer with a considerably broader molecular weight distribution (*Đ* = 1.9, Table 2) that was devoid of PPS (red NMR spectrum, Figure 2B, bottom).

**Table 2:** Physico-chemical Characterization of RAFT Polymers

		theoretical			^1^H NMR^d^				
		monomer equiv^b^			monomer equiv			GPC^e^	
PMA used^a^	polymer name	DMA	DEA	*M*_n_ (Da)^c^	DMA	DEA	*M*_n_ (Da)	*Đ*	*M*_n_ (Da)
BB-PMA	PSM(BB)	100		13400	90		12400	1.15	10600
BPA-PMA	PSM(BPA)	100		13700	82		11900	1.08	11800
BP-PMA	PSM	100		13200	90		12200	1.18	13700
BP-NEG	PSM(NEG)	100		12700	90		9000 (12100)^f^	1.95	11000
BP-PMA	PSM3E1	75	25	14000	68	22	12900	1.18	13600
BP-PMA	PSM1E1	50	50	14700	44	44	13300	1.20	12600
BP-PMA	PSM1E3	25	75	15400	25	72	15000	1.17	12800
BP-PMA	PSE		100	16100		90	14800	1.22	11800

a The first four rows refer to DMA RAFT homopolymerization conducted with three different PMAs and a negative control (BP-NEG); the second four rows refer to DEA homopolymerization and DEA/DMA copolymerization using BP-PMA.

b 50 monomers per arm, i.e., 100 monomers per (difunctional) PMA.

c Calculated assuming a quantitative monomer conversion.

d The monomer equivalents are calculated by estimating the monomer conversion via the decrease in the integral of the resonance at 5.6 ppm (acrylamide double bond), after normalization against the trioxane resonance at 5.1 ppm and expressed as the equivalents of monomers theoretically attached to every PMA. The *M*_n_ is obtained by summing the weight of these monomer equivalents with that of the PMA.

e Measured through triple-detection GPC analysis in DMF; GPC traces are provided in Supporting Information, Figure S14.

f Since signals of BP-NEG are almost completely absent in the spectrum, we here report the *M*_n_ value calculated without considering the BP-NEG contribution, and in brackets the value obtained by taking that into account.

We note the following:

(A) The character of the DMA polymerization with BP-NEG is quite different from the others and did not lead to appreciable integration of DMA with the polysulfide chains, as on the contrary was observed for the other PMAs. In short, we can confirm that the latter do polymerize with a RAFT and not a free radical mechanism.
(B) The inhibition period was considerably longer in BPA-PMA than in BB-PMA and BP-PMA. This seems to suggest that the overall behavior of these PMAs is ascribed to the nature of the leaving radicals.

We ascribe the long inhibition period of BPA-PMA to the low reactivity/reinitiation rate of the leaving BPA radical, which indeed is not one of the most commonly used RAFT agents,^34^ due to its stability. As a result, we chose BP-PMA for all further polymerizations, because of its higher monomer conversion rate.

### Colloidal Self-Assembly

**Figure 3:**
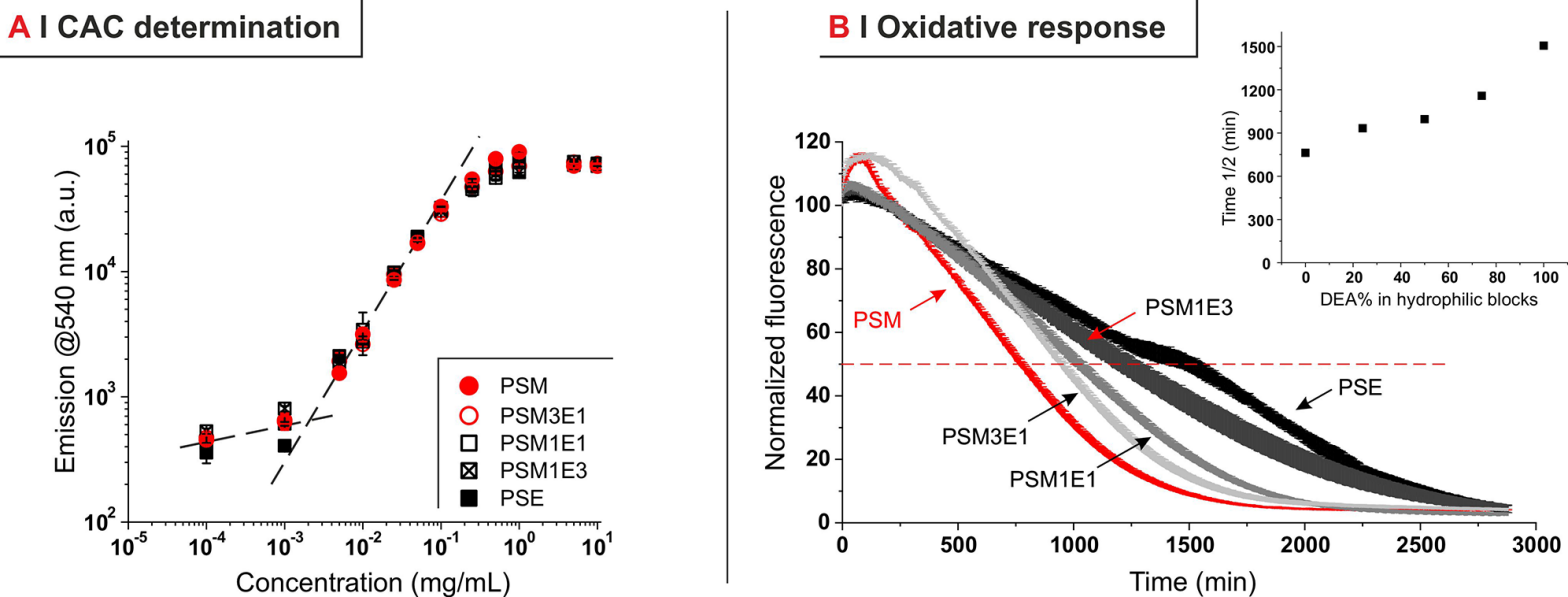
(A) CAC was defined as the polymer concentration at which the fluorescence of 1.5 × 10^–6^ M Nile Red quenched in water starts recovering; no significant difference is observed among the five polymers (*n* = 3). (B) Oxidation experiments were performed on block copolymer dispersed in deionized water at a concentration corresponding to 5 mM of thioethers and loaded with Nile Red; the oxidation by 4 wt % H_2_O_2_ is followed by monitoring the progressive quenching of Nile Red fluorescence by the ingress of water (negative controls showing no effect of atmospheric oxygen are provided in Supporting Information, Figure S16). The inset shows the time at which the fluorescence decreased to half of its original value (*t*^1/2^) as a numerical indicator of the kinetics (*n* = 3).

In water, all these amphiphilic triblock polymers produced colloidal structures with a size significantly below 100 nm (40–50 nm for all except PSE, Table 3) and with significantly low values of critical aggregation concentration (CAC; Table 3 and Figure 3A). The latter finding indicates a good stability of these structures against dilution, which, in perspective, is a critical attribute for parenteral administration: a high CAC implies that a moderate extent of dilution may solubilize the aggregates; clearly, interactions with, e.g., blood-borne biomolecules may complicate this framework, but PDMA is not prone to protein adsorption.^35^ Importantly, CAC values were independent of the DMA/DEA ratio, which shows the aggregation to be solely influenced by the PPS block, at least at room temperature. Of note, PDIs are relatively high, although below what typically is defined as polydisperse;^36^ this may relate to the presence of some larger (and out-of-equilibrium) aggregates, but their large values are also due to the small size of these colloids: the peak half-widths are <40 nm for all polymers bar PSE.

**Table 3:** Colloidal Characterization of Self-Assembled Structures

polymer [full name]	hydr. size (nm)^a^	PDI^a^	“dry” size (nm)^b^	CAC (mg/mL)^c^
PSM [PDMA_45_-*b*-PPS_37_-*b*-PDMA_45_]	60	0.5	20 ± 3	0.02
PSM3E1 [P(DMA_34_-*ran*-DEA_11_)-*b*-PPS_38_-*b*-P(DMA_34_-*ran*-DEA_11_)]	50	0.5	37 ± 3	0.02
PSM1E1 [P(DMA_22_-*ran*-DEA_22_)-*b*-PPS_38_-*b*-P(DMA_22_-*ran*-DEA_22_)]	40	0.4	34 ± 3	0.02
PSM1E3 [P(DMA_13_-*ran*-DEA_36_)-*b*-PPS_38_-*b*-P(DMA_13_-*ran*-DEA_36_)]	40	0.5	31 ± 2	0.02
PSE [PDEA_45_-*b*-PPS_39_-*b*-PDEA_45_]	90	0.6	24 ± 3	0.02

a *Z*-average hydrodynamic size and polydispersity index (PDI) at 25 °C, from DLS measurements on 1 mg/mL dispersions.

b Numerical average of the size of the aggregates from TEM images of dried aqueous dispersions of polymers (see Supporting Information, Figure S15). The large difference between these values and those of hydrodynamic size is due to the swelling of the hydrophilic blocks in water.

c The fluorescence of encapsulated Nile Red (constant concentration) progressively decreases upon dilution of its polymer dispersions. Please note that the relatively generous size of these colloidal structures and their equally broad dispersity in size may indicate that the polymer aggregates are not micellar in nature; hence it is here preferred to describe them simply as aggregates, hence the avoidance of a term such as critical micellar concentration (CMC).

PPS blocks determine not only the hydrophobically driven assembly but also the oxidative response; however, the kinetics of the latter was also affected by the composition of the hydrophilic blocks (Figure 3B): PSM (PDMA homopolymeric blocks) reacted about two times more rapidly than PSE (PDEA homopolymeric blocks), and the reactivity of the copolymers was inversely proportional to their DEA content (inset in Figure 3B). This effect may be due to the solubility of H_2_O_2_ in the outer shell, which, by decreasing with increasing content of the less polar DEA, would determine a slower ingress of the oxidant into the PPS core.

**Figure 4:**
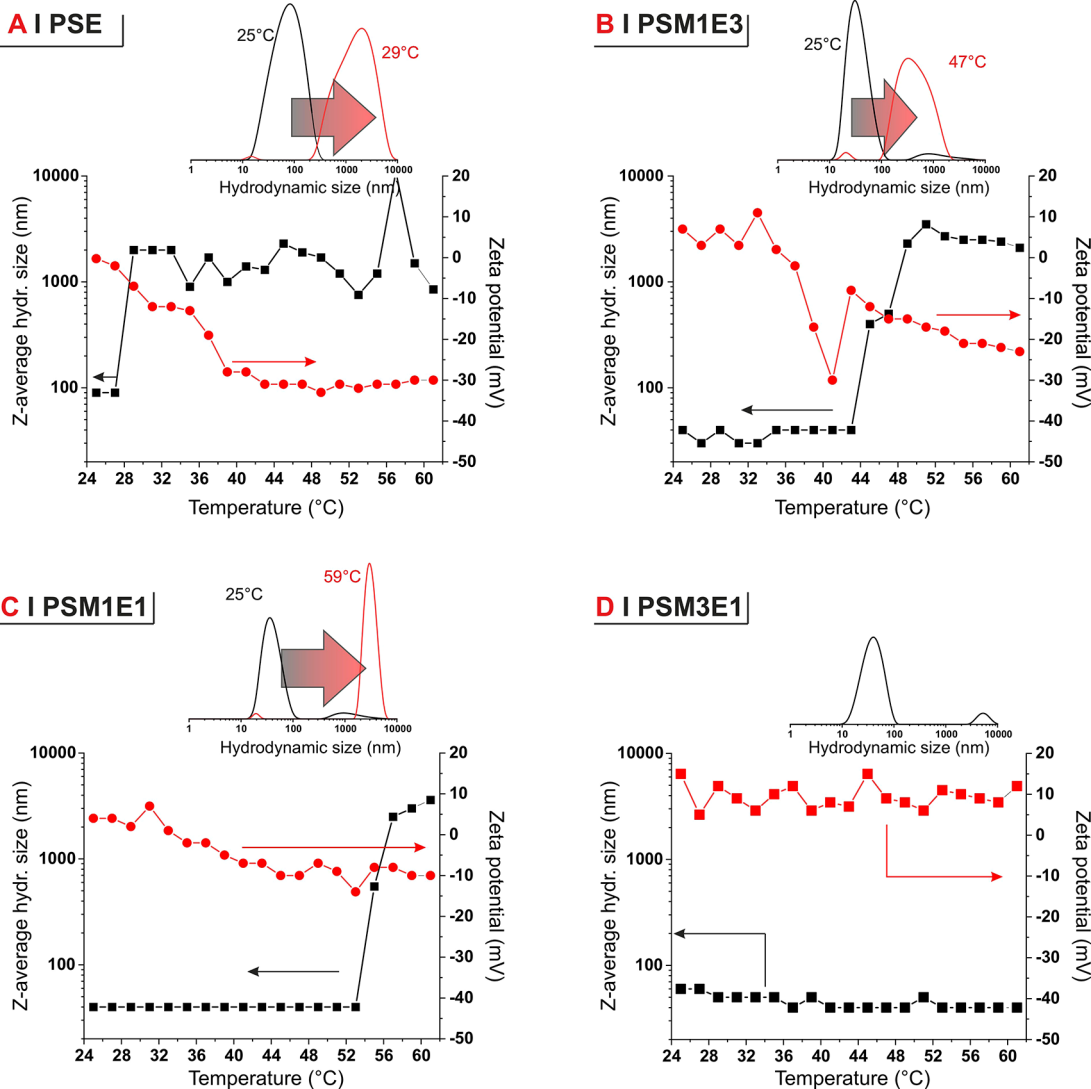
*Z*-average hydrodynamic size (black squares, left axis) and zeta potential (red circles, right axis) as a function of temperature, upon heating in the 25–60 °C temperature range for PSE and the three PSM_*x*_E_*y*_ copolymers, all at a 1 mg/mL concentration in deionized water. PSM3E1 (as well as PSM, data not shown) exhibits no responsiveness in this temperature range. Please note that higher temperatures were not investigated, as on the one hand this is not physiologically relevant and on the other hand the determination is also largely affected by water evaporation and the polymer concentration is not constant throughout the experiment.

The DEA/DMA ratio had an even more dramatic effect on the temperature dependency of the colloidal size; aggregation at >1 μm scale was recorded above 28 °C for 1 mg/mL PSE, which is in line with literature values for the transition temperature of PDEA-based systems.^26,27,30^ This transition, however, moved above 44 °C for PSM1E3, above 52 °C for PSM1E1 (Figure 4), and out of the temperature range studied (up to 60 °C) for polymers with a higher DMA content. The transition temperature can therefore be tuned upward by decreasing the DEA/DMA content in the random hydrophilic (co)polymer blocks. Of note, the transition temperatures are almost identical to those recorded for DMA/DEA copolymers obtained via free radical polymerization,^31^ which confirms that the PPS block has no significant effect on this phenomenon.

In short, at the cell culture/body temperature of 37 °C, PSE formed large, micrometer-sized aggregates (likely with poorly hydrated hydrophilic coronas), while all other polymers assembled in <100 nm colloids. It is noteworthy that the extent of PSE aggregation markedly depended on concentration. For example, at 1 mg/mL a transition can only be recorded via DLS (increase in size at 28–30 °C, Figure 4A) but samples did not become visibly turbid, suggesting that aggregates were not excessively large. On the other hand, at concentrations between 10 and 200 mg/mL (see Supporting Information, Figure S17) it was possible to see well-defined cloud points in the region 37–42 °C, which indicate the formation of much larger aggregates; at even higher concentrations, macroscopic gelation was observed (above 200 mg/mL; seen via vial inversion or shear rheometry, see Supporting Information, Figure S18).

In summary, while these polymers are similar in terms of ROS scavenging activity and micellar assembly, the composition of the hydrophilic shell (the DEA content) determines their overall morphology: stable micelles for PSM and PSM3E1, large aggregates for PSE, and likely dynamic micelles (close to an aggregation transition) for the intermediate PSM1E1 and PSM1E3l.

**Figure 5:**
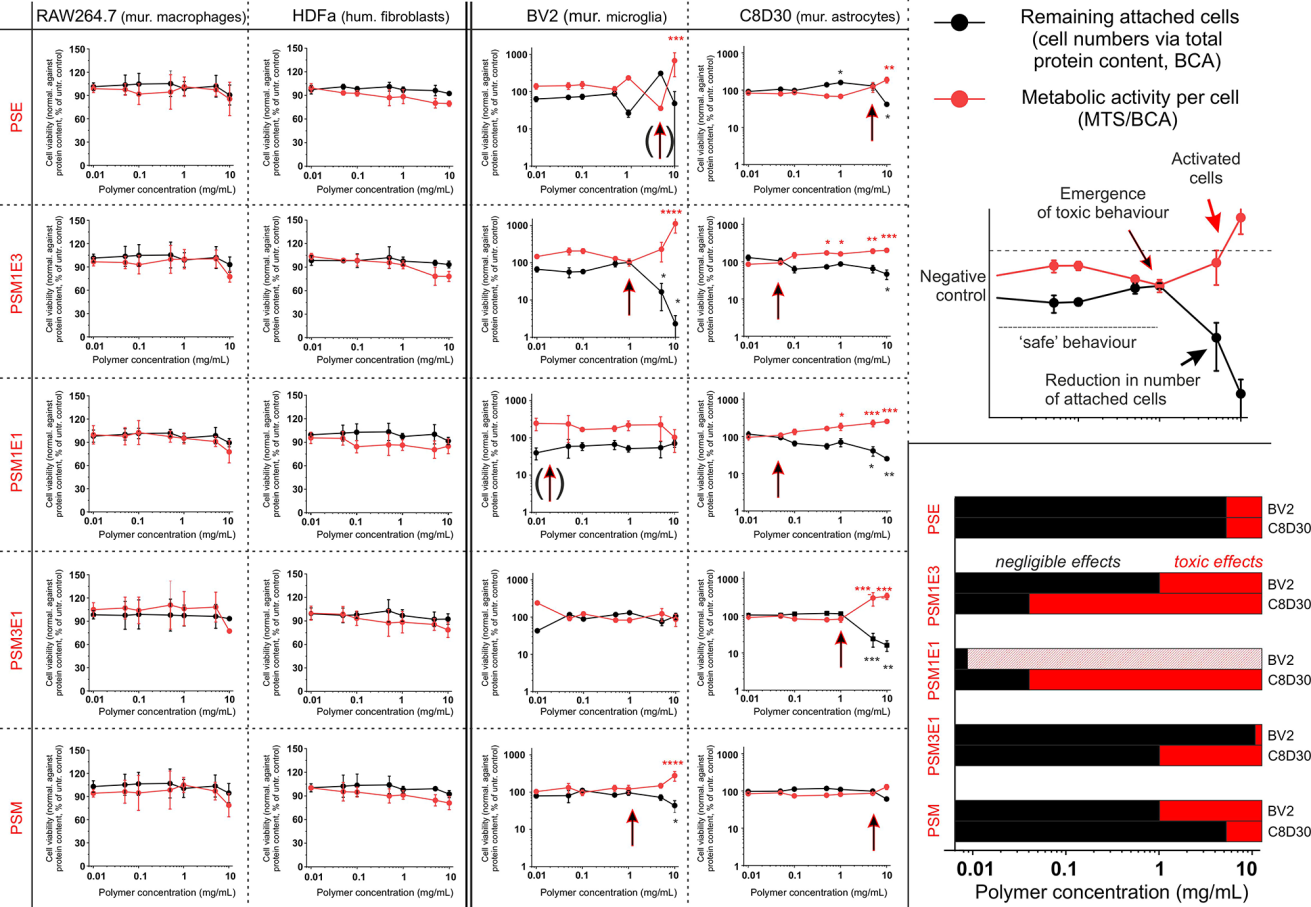
(left and middle) Four cell lines were exposed to 0.01–10 mg/mL dispersions of the five polymers for 24 h, measuring the number of attached cells (assessed through the total protein content; BCA assay, black symbols), which decreases with significant cell death (detachment of cell bodies), and the average cellular mitochondrial reductase activity (MTS assay, normalized against the protein content, red symbols). The latter indicator may be either increased (activation, e.g. preapoptosis) or decreased (shutting-down of the whole cell metabolism) by an unwelcome and potentially toxic input. (right) Top graph shows how the point where cell number (decreasing) and mitochondrial activity (increasing) cross can be used as the limit of the “safe” polymer concentrations; these two indicators allow a much more sensitive determination of the “safe”/healthy zone than MTS alone (see Supporting Information, Figure S19). The bottom graph summarizes the “safe” areas (black), the clearly toxic ones (red) and those showing moderate activation (red stripes). Please note that MTS-based IC50 could not be calculated, since non-normalized MTS did not reach 50% of the negative controls for all the polymer/cell combinations except PSM1E3 on BV2. Black arrows with red contour indicate the start of toxic behavior. Arrows between parentheses means that the toxicity onset is not clearly determined (*n* = 3–5).

To ascertain whether these morphological differences may impact cell viability, we have employed four well-established cell lines, monitoring the number of attached cells (number of cells proportional to protein content; BCA assay, black dots/lines in Figure 5) and the average cellular mitochondrial activity (MTS normalized against protein content; MTS/BCA, red dots/lines in Figure 5). Murine RAW macrophages and human dermal fibroblasts (HDFa) showed no effect after a 24 h exposure to any of the polymers, for concentrations up to 10 mg/mL (left part of Figure 5), which indicates all these materials to be benign in nature.

BV2 microglia and C8D30 astrocytes are significantly more sensitive cellular models and, although always within a framework of very low toxicity, showed a more nuanced perspective. In this case, we take the point where metabolic activity and viability, respectively, start increasing and decreasing (arrows in Figure 5) as where cellular stress becomes significant. Below 1 mg/mL, only PSM1E3 and PSM1E1 caused cellular stress, and of moderate intensity (middle and right part of Figure 5). We previously noted that at 37 °C PSM1E3 and PSM1E1 are not too distant from their cloud point, thus dynamic association through DEA residues, potentially allowing interactions with cell membranes, may be the cause.

**Figure 6:**
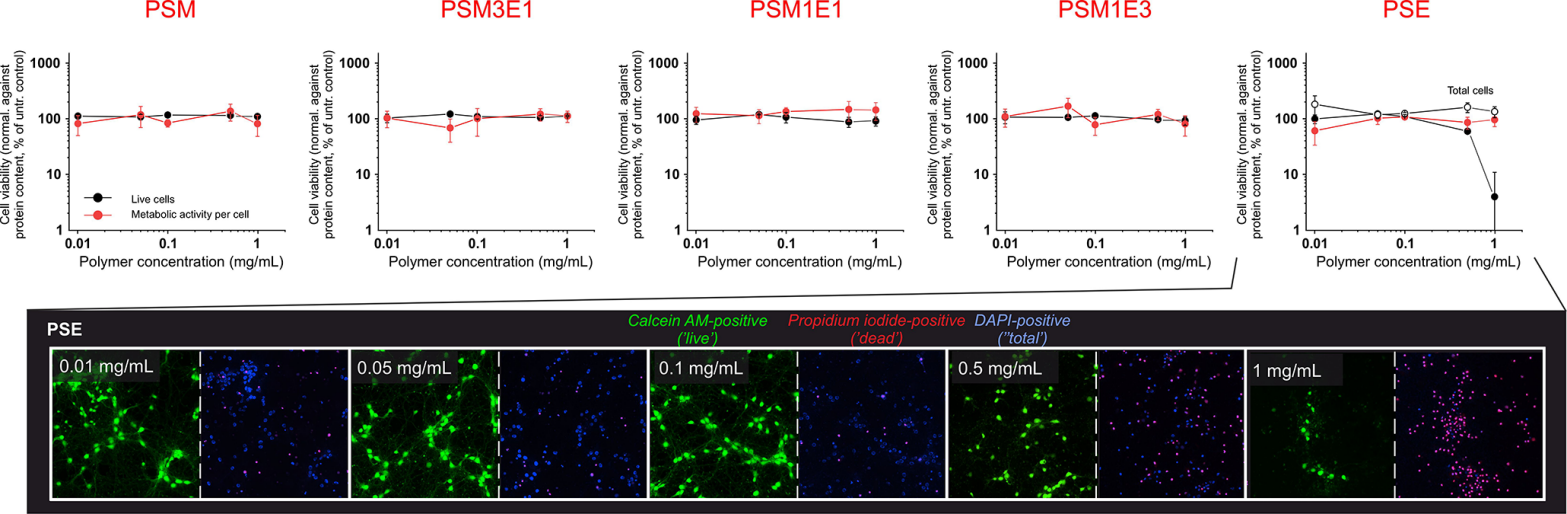
(top) Potential toxic effects of 24 h exposure of primary neurons to the polymers were evaluated by quantifying the average mitochondrial activity per cell (MTT divided by the total number of attached (DAPI-positive) cells; red circles) and the actual numbers of attached (DAPI-positive) and live (calcein-positive) cells. For all polymers except PSE, the last two data sets were always virtually identical, and only the % of calcein-positive cells is reported (black circles); when exposed to high concentrations of PSE, however, a significant number of cells were positive to DAPI and not to calcein (but to propidium bromide), therefore both calcein-positive cells (black circles) and attached cells (empty circles) are reported to highlight this behavior. (bottom) Fluorescence microscopy pictures of primary neurons exposed for 24 h to different concentrations of PSE (pictures for all other polymers are provided in Supporting Information, Figure S20) showing that, despite all cells being still attached, at 1 mg/mL the vast majority are unable to convert calcein, although the average mitochondrial activity per attached cell (above) is unchanged.

Above 1–5 mg/mL, signs of stress were caused by most polymers, and PSM1E3 also markedly decreased cell viability; therefore 1 mg/mL was chosen as the maximum concentration for all further cell experiments, e.g., those with the more sensitive primary cortical neurons. Their average mitochondrial activity per cell (Figure 6) and the number of attached cells were not affected by the presence of PSM and of any copolymer, confirming their “safe”/stress-free character up to at least 1 mg/mL.

Differently, PSE increased the fraction of propidium bromide-positive (dead) cells and reduced that of calcein-positive ones for concentrations ≥0.5 mg/mL. Curiously, the neuron mitochondrial activity remained largely untouched, despite the extensive membrane damage (i.e., propidium iodide staining) and the loss of active cytoplasmic enzymatic activity (calcein). This behavior, quite different from, e.g., that of the rather similar PSM1E3, may be due to PSE being the only polymer actually producing pretty large aggregates, which would sediment on top of the cells, both creating a physical barrier to nutrient diffusion and artificially increasing the local concentration of polymer. This indicates that thermal aggregation may actually lead to detrimental effects, e.g., limiting the advantageous performance of PPS-based materials as demonstrated in the CNS.^15,37^ Of note, *in vivo* PSE may aggregate differently, therefore the above should not be taken as an actual forecast of the potential toxicological profile of the material.

With this *caveat* in mind, we have evaluated the ROS scavenging-based anti-inflammatory activity, using three cellular models of neuroinflammation: LPS-activated microglia and astrocytes and neurons activated using microglia-conditioned medium, MCM, which allows one to avoid the direct use of the rather toxic LPS on these more sensitive cells.^38,39^

**Figure 7:**
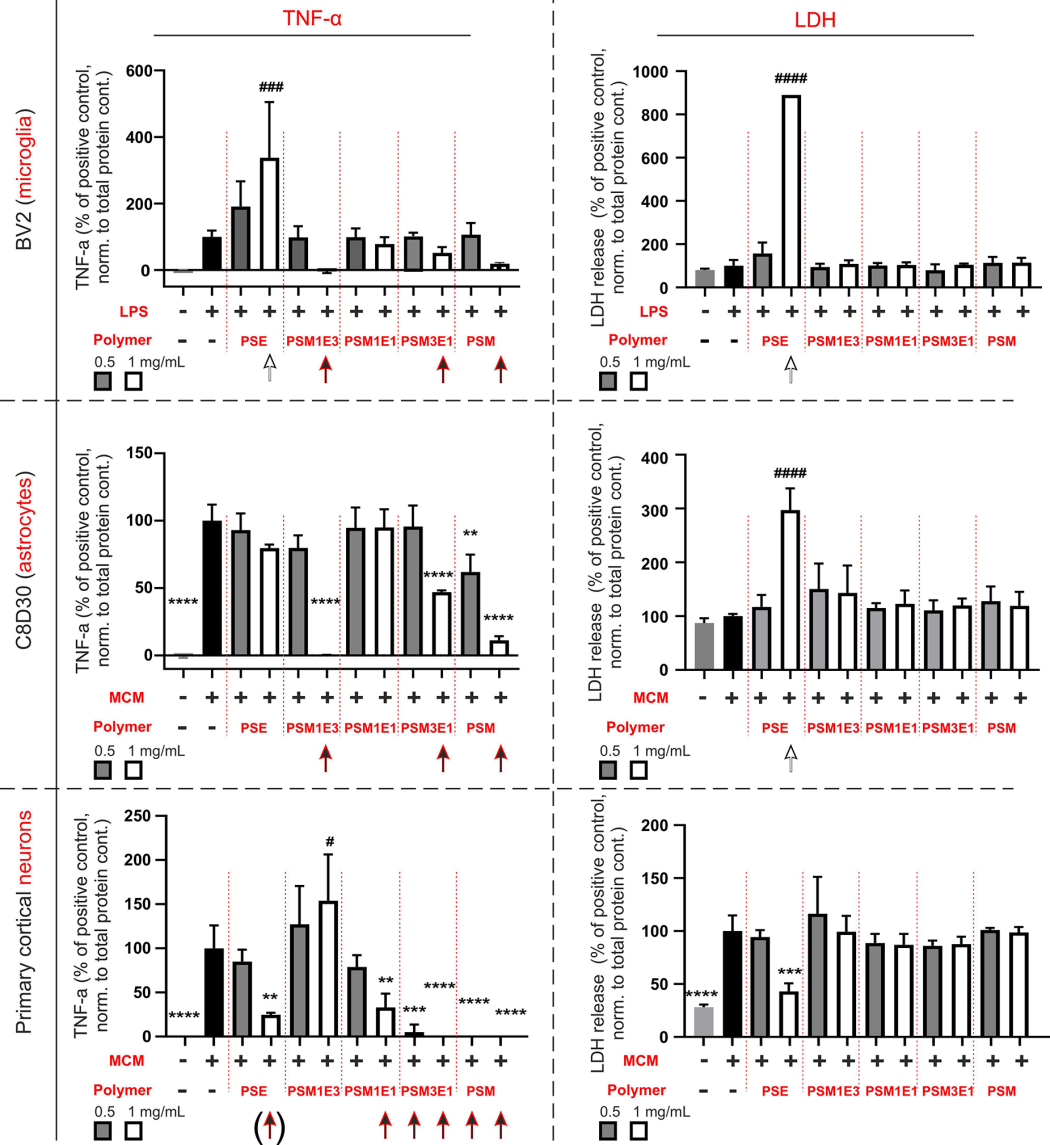
Release of TNF-α (left) and of LDH (right) in the supernatant, as markers of inflammatory activation and membrane damage, respectively, after 24 h activation with inflammatory stimuli (0.5 μg/mL for microglia, and MCM for astrocyte and primary neurons) and 24 h co-incubation with polymers (0.5 or 1 mg/mL) and inflammatory stimuli. Please note that the data are divided by the protein content (i.e., the values are TNF-α and LDH “per cell”) and then against the positive controls (no polymer, reported as 100%).

The following observations can be made:

(A) In these three cellular models of CNS inflammation, activation coincides with the release of the pro-inflammatory cytokine TNF-α (graphs in the left part of Figure 7) and sizable membrane damage, as witnessed by the release of LDH (graphs in the right part of Figure 7).
(B) At 1 mg/mL, PSE was detrimental: it increased the release of LDH in activated astrocytes and microglia and in the latter also increased TNF-α production (white arrows in Figure 7). A higher release of LDH or TNF-α was not recorded on cortical neurons, which may appear surprising if one considers the membrane damage making them propidium iodide-positive. However, one must note that the data in Figure 7 are normalized to cell number, which for PSE treatments differs significantly from that of calcein-positive cells.
(C) Treatment with any other polymer (0.5 or 1 mg/mL) did not induce any further LDH release, and at 1 mg/mL almost always significantly reduced the release of TNF-α (red arrows in Figure 7). The effect was remarkable with neurons, whose production of the inflammatory cytokine was brought back to homeostatic levels already at 0.5 mg/mL of PSE1M3 or PSM. As an additional proof of the anti-inflammatory effects of this family of polymers, the levels of TNF-α were reduced despite neurons showing signs of significant membrane damage (elevated levels of LDH).

In summary, using a panel of cells that recapitulates the cellular diversity of the CNS, this family of polymers showed remarkably low toxicity and promising anti-inflammatory activity, above all on inflamed neurons. The main *caveat* is that the capacity of large-scale, PDEA-mediated (thermoresponsive) aggregation at the cell culture temperature appears to be detrimental to delicate cells such as primary cortical neurons. A decrease in viability is also apparent for ≥0.5 mg/mL PSE (Figures 6 and 7), and noticeable, although at best mild, for >1 mg/mL PMS1E3 and PMS1E1 (Figure 5); this appears to confirm the link between capacity of aggregation and undesired side effects and therefore also suggests a potentially antagonistic (at least for PSE) effect with the polymer’s own ROS scavenging and anti-inflammatory action. Therefore, we showed that, on the one hand, non-aggregating polymers displayed a remarkable ability to ameliorate neuroinflammation, which lends considerable mechanistic credence to other *in vivo* examples of CNS-based applications of polysulfides, e.g., in the management of inflammation caused by ischemic stroke or traumatic brain injury^40^ or in the enhancement of the brain survival of neural progenitor cells.^37^ On the other hand, large-scale aggregation may be detrimental, e.g., via membrane damage (ethidium bromide assay) by exposed DEA residues, although we cannot rule out an effect on, e.g., oxygen and nutrient permeation by the aggregates sedimenting on the cell layers. Of note, the latter effects may be specific to CNS cells: the lack of toxicity on 3T3 fibroblasts and RAW macrophages may suggest applicability to other less sensitive *loci* within the body.

## Conclusion

In this study, we have optimized the synthetic conditions to obtain well-defined oxidation-sensitive amphiphilic block copolymers via successive anionic and RAFT polymerization. This has allowed us to prepare a family of polymers virtually identical in primary structure, molecular weight distribution, and micellization behavior but differing in the composition of the hydrophilic shell. This parameter (the DEA/DMA ratio) affected the kinetics of the oxidative response: the more hydrophilic, the more rapid the response, although the differences were not huge and indeed all polymers exhibited anti-inflammatory behavior on three different activated cellular models.

The hydrophile composition had a more dramatic effect on the capacity of micellar dispersions to undergo large-scale aggregation: only high-DEA polymers showed temperature-induced transitions potentially leading to a macroscopic aggregation. This feature, on the one hand, is potentially beneficial allowing the *in situ* formation of localized depots at specific body locations upon injection of appropriately cold formulations. On the other hand, although this family of polymers showed no toxicity in classical cell lines (3T3 fibroblasts and RAW macrophages) and limited effects in astrocytes and microglial cell lines (C8D30 and BV2s, respectively), the thermoresponsive, depot-forming PSE (PDEA-based) presented clear toxicity in clinically relevant primary cortical neurons.

Although this effect may be affected by the environmental constraints of 2D cultures (sedimentation of aggregates), it highlights the necessity of exercising caution in the use of depot-forming systems for brain delivery. Having said this, all other micellar polysulfides, not aggregating at physiological temperatures, were capable of blocking neuroinflammation in microglia, astrocytes, and primary cortical neurons, which makes them attractive for further biomedical investigations.

## Supporting Information Available

Additional synthesis and characterization data including ^1^H NMR and MALDI-ToF spectra, GPC curves, and microscopy, as well as numerical tables for methods, optimization, and monomer conversion (PDF)
