# Supplementary material for: Dual Thermal- and Oxidation-Responsive Polymers Synthesized by a Sequential ROP-to-RAFT Procedure Inherently Temper Neuroinflammation
Source: Biomacromolecules. 2023 Feb 9;24(10):4478–93. doi: 10.1021/acs.biomac.2c01365 (PMC10565819; doi:10.1021/acs.biomac.2c01365)
Supplement: Supplementary file 1 — bm2c01365_si_001.pdf [file bm2c01365_si_001.pdf]

Dual thermal- and oxidation-responsive polymers synthesized by a sequential ROP-to-RAFT procedure inherently temper neuroinflammation

Zulfiye Y. Turhan[1,2], Richard d'Arcy[2,§], Farah El Mohtadi[1,‡], Nora Francini[2, §], Mike Geven[2], Valentina Castagnola[3,4], Aws Alshamsan[5,6], Fabio Benfenati[3,4], Nicola Tirelli[1,2*]

[1] Division of Pharmacy and Optometry, School of Health Sciences, University of Manchester, Oxford Road, Manchester M13 9PT, United Kingdom
[2] Laboratory for Polymers and Biomaterials, Fondazione Istituto Italiano di Tecnologia, 16163 Genova, Italy
[3] Center for Synaptic Neuroscience and Technology, Fondazione Istituto Italiano di Tecnologia, 16132 Genova, Italy
[4] IRCCS Ospedale Policlinico San Martino, 16132 Genova, Italy
[5] Department of Pharmaceutics, College of Pharmacy, King Saud University, P.O. Box 2457, Riyadh 11451, Saudi Arabia; aalshamsan@ksu.edu.sa
[6] Nanobiotechnology Unit, College of Pharmacy, King Saud University, P.O. Box 2457, Riyadh 11451, Saudi Arabia

* Correspondence: nicola.tirelli@iit.it

§ Current address: Department of Biomedical Engineering, Vanderbilt University, Nashville, TN 37235, USA
‡ Current address: Faculty of Science & Health, School of Pharmacy & Biomedical Sciences, University of Portsmouth, White Swan Road, St. Michael's Building, Portsmouth PO1 2DT, United Kingdom

SUPPORTING INFORMATION

1SI. Additional preparative operations

1.1SI Synthesis of the protected bifunctional initiator (DOTAc).

7.6 g (83.2 mmol of thiols) of 3,6-dioxa-1,8-octane-dithiol (DOT) and 25.2 g (249.6 mmol, 3 equiv per SH) of triethylamine were introduced into 400 mL of dried THF under argon. The reaction flask was transferred to an ice bath and a chilled solution of 11.8 mL (13 g, 165.6 mmol, 2 equiv per SH) of acetyl chloride in 50 mL of THF was added dropwise under vigorous stirring. The mixture was allowed to warm up to room temperature and react for a further 4 h, then filtered and the volatiles were removed via rotary evaporation. The residue was diluted with 100 mL of dichloromethane, washed 3 times with brine, dried over Na_2SO_4, filtered and finally concentrated in vacuo to yield an oil that was purified by column chromatography using hexane:ethyl acetate (3:1) as the mobile phase and silica gel as the stationary phase. 6.7 g (88% yield) of a clear-yellow oil was obtained.

^{1}H NMR ($CDCl_3$): δ = 2.35 (s, 6H –CH_2–O–CH_2–CH_2–S–C(=O)–CH_3), 3.10 (t, 2H –CH_2–O–CH_2–CH_2–SC(=O)–CH_3), 3.61 (t, 2H –CH_2–O–CH_2–CH_2–S–C(=O)–CH3, 3.62 ppm (t, 2H –CH_2–O–CH_2–CH_2–S–C(=O)–CH_3).

^{13}C NMR ($CDCl_3$): δ = 28.8 (s, –CH_2–O–CH_2–CH_2–S–C(=O)–CH_3), 30.5 (s, –CH_2–O–CH_2–CH_2–SC(=O)–CH_3), 69.8 (s, –CH_2–O–CH_2–CH_2–S–C(=O)–CH_3, 70.2 (s, –CH_2–O–CH_2–CH_2–S–C(=O)–CH_3), 195 ppm (s, –CH_2–O–CH_2–CH_2–S–C(=O)–CH_3).

FT-IR (ATR); $\bar{\nu}$ = 2928 (ν_{as} methylene C–H), 2865 (ν_s methylene C–H), 1686 (ν C=O), 1099 (ν_{as} C–O–C), 622 cm^{-1} (ν C–S).

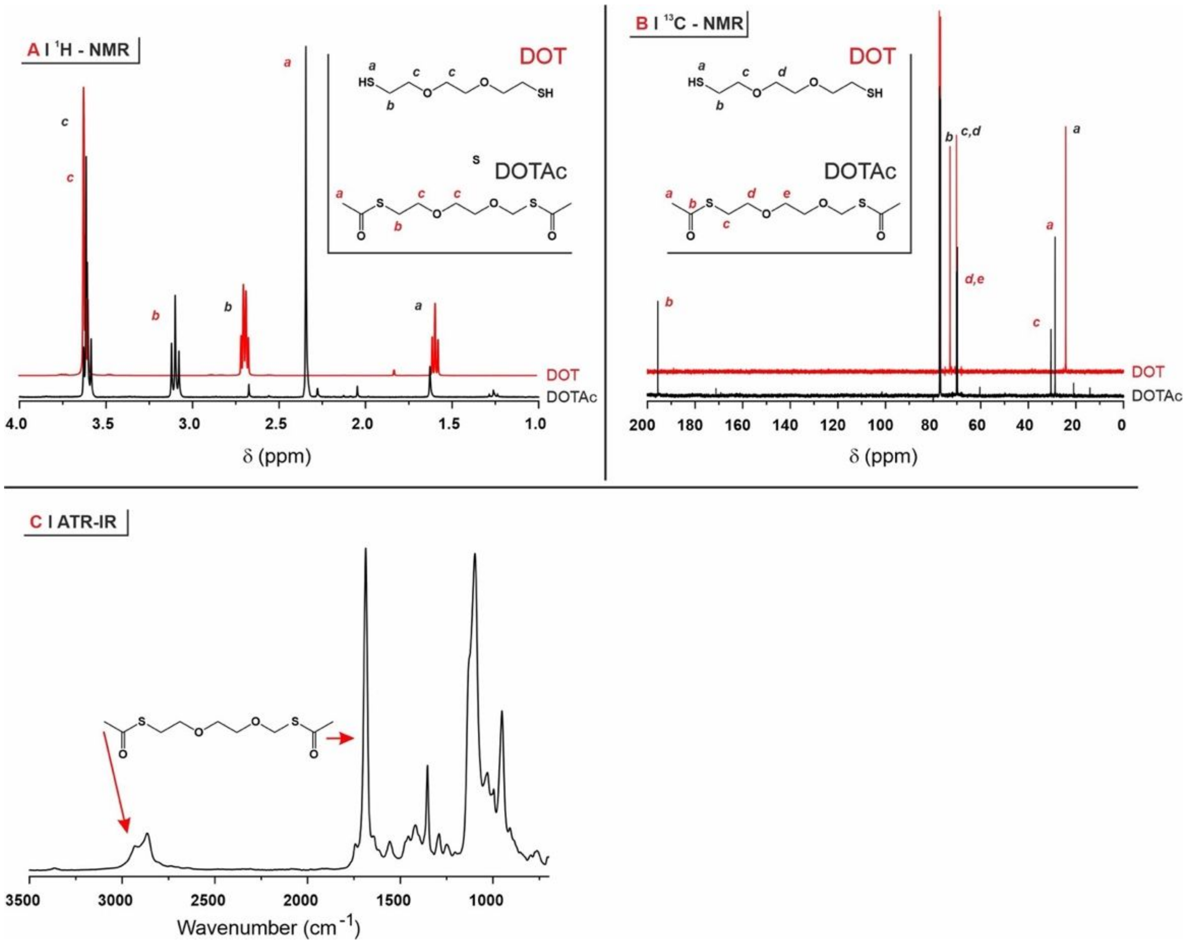

Figure S1. ***A.*** ^{1}H and ***B.*** ^{13}C NMR spectra of DOT (red spectra, black assignments) and DOTAc (black spectra, red assignments). ***C.*** IR spectrum of DOTAc. Red arrows point to the absorptions related to the stretching vibrations of methyl (□as at 2865 cm^{-1}) and carbonyl groups (1686 cm^{-1}).

1.2SI Synthesis of propylene sulfide (PS)

460 g (6 mol, 1.25 equiv per propylene oxide (PO)) of ammonium thiocyanate was dissolved in 800 mL of deionized water obtaining a concentration of 0.58 g/mL (7.6 M) and the resulting solution was degassed with argon gas for 1 hour. It was thereafter introduced in an Atlas HD reactor (Syrris Ltd., Royston, United Kingdom), comprising a jacketed reactor, jacket, and

reactor temperature probe, 50W heating probe, overhead stirrer, and an inlet for dry argon gas. Under an argon atmosphere, 400 mL of degassed DCM was added while stirring, to form an emulsion with a 2:1 water:DCM (v/v) ratio. 280 g (4.8 mol) of PO was subsequently added continuously via a dropping funnel at a rate that allowed the reactor setup to control the temperature between 28 and 32 ˚C (2.3 mL/min in our setup). The final PO concentration with respect to the aqueous phase was 6 M (0.35 g/mL). The reaction was left to run for 16 hours after initial PO addition. The organic phase was thereafter distilled out of the reactor under reduced pressure (300 mbar) and at 35 ˚C, collecting it in a dry ice-cooled flask. The distillate was dried over Na_2SO_4 followed by fractional distillation to separate DCM and PS between 80 ˚C (DCM-rich phase, confirmed by ATR-IR) and 120 ˚C (PS-rich phase) under an argon atmosphere. The average yield was 58% by weight, with an average purity of 99 mol% (only impurity being DCM). ΔH= 47±3.4 kJ/mol.

^{1}**H NMR** ($CDCl_3$): δ = 1.54 (d, CH_3), 2.14 (d, 1 diastereotopic CH_2), 2.53 (d, 1 diastereotopic CH_2), 2.94 ppm (m, CH).

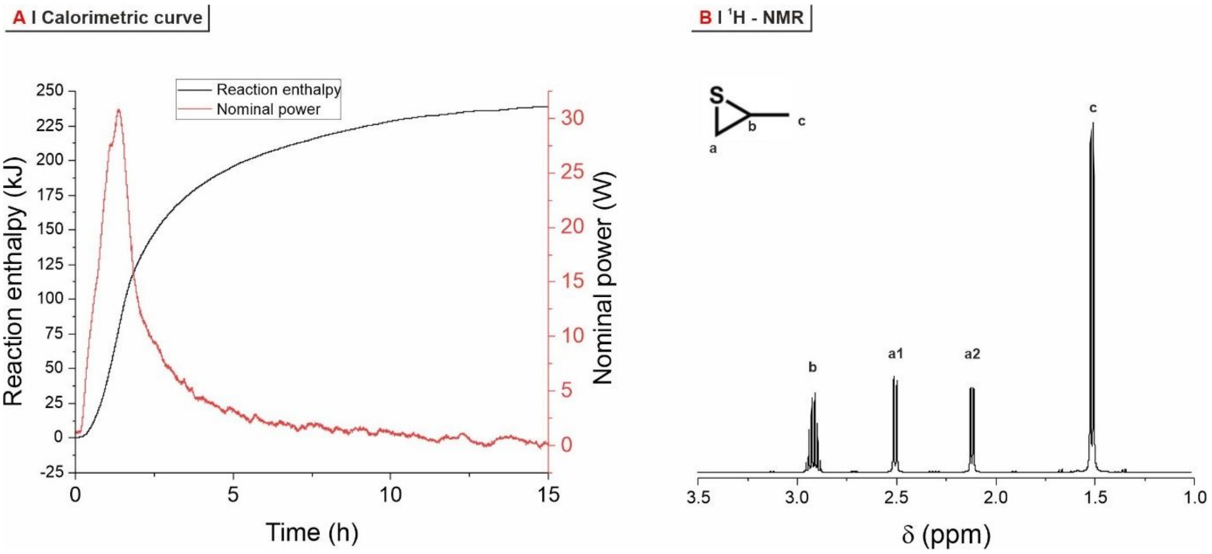

Figure S2. ***A.*** *In situ* calorimetric curve during the reaction. ***B.*** ^{1}H NMR spectrum of PS.

1.3SI Optimization of end-capping conditions

Six potential RAFT-active end-cappers were employed: benzyl bromide (BB, a primary halide), ethyl 2-bromopropionate (BP), ethyl α-bromophenylacetate (BPA), 2-bromopropionamide (BPAM), 2-bromopropionitrile (BPN)) and ethyl α-bromoisobutyrate (BIB). The data are summarized in Table S1 and the procedures leading to quantitative end-capping and monomodal molecular weight distributions (use for further experiments) are highlighted in red. The general experimental procedures are always those described in the main text, except for the parameters mentioned in Table S1. Notes:

A) **BPAM**. In run 23, the ^{1}H NMR spectrum indicated quantitative end-capping, the **C**=S signal was missing in the ^{13}C NMR spectrum and GPC chromatogram showed multimodal distribution. By doubling all reagents (TBP, CS_2 and BPAM) as in run 25, GPC secondary

peaks were reduced, and by further replacing Na with DBU (as the thiolate counterion in the end-capping) as in run 26, the **C**=S signal appeared in the ^{13}C NMR spectrum. However, in no experiment monomodal MW distribution were obtained and this end-capper was therefore abandoned.

B) **BPN**. The end-capping was not successful when using Na as a counterion (runs 19, 21, 22), but also when DBU was used instead, a multimodal MW distribution was observed (run 20). Therefore, BPN was also excluded from further evaluation.

C) **BIB**. End-capping tried in THF at room temperature was completely unsuccessful with multimodal MW distribution (data not shown). The reaction was therefore attempted in DMF, adding about 10% degassed water, and using longer reaction times, all factors thought to promote the SN_1 reaction of the sterically hindered end-capping agent. However, quantitative end-capping was not achieved in any of the conditions assessed (runs 26-29).

D) **BB, BP, and BPA**. For these monomers, conditions were found to obtain quantitative end-capping and monomodal MW distribution. For **BB** (benzylic halide), this meant a 1: 1.5 : 4 : 5 thiol/TBP/CS_2/BB molar ratio (run 2). For **BPA** (benzylic and electron-poor, but also very sterically hindered), the same conditions yielded a multimodal distribution (run 12), and a monomodal one could only be obtained using double the concentration of all reagents (run 15). For **BP** (secondary bromide), under most conditions the end-capping was relatively

satisfactory, but it was only using higher excesses of reagents (1: 3 : 8 : 10 thiol/TBP/CS_2/BP molar ratio), double concentration of all and DBU as a counterion that a monomodal MW distribution was obtained.

Table S1. Reaction conditions for the end-capping of PPS with RAFT-active groups

	End-capper	Solvent	PS conc. [a] (mg/mL)	Eq.s to thiols			Thiolate counterion [b]	Temp. (°C)	Duration	End-capp.yield (mol%) [c]	MW distrib. [d]
				TBP	CS_2	Bromide					
1	BB	THF	85	1.5	3	3	Na	RT	2h	80	mono
2	BB	THF	85	1.5	4	5	Na	RT	overnight	100	mono
3	BP	THF	85	2.5	3	3	Na	RT	5h	= [e]	[f]
4	BP	THF	85	1.5	4	5	Na	RT	5h	100	multi
5	BP	THF	170	3	8	10	Na	RT	5h	90	multi
6	BP	THF	170	5	13.5	16.5	Na	RT	5h	98	multi
7	BP	THF	170	3	10	10	Na	RT	5h	94	multi
8	BP	THF	170	3	8	12	Na	RT	5h	83	multi
9	BP [h]	THF	170	3	8	10	Na	RT	5h	66	multi
10	BP	THF	170	3	8	10	DBU [g]	RT	5h	100	mono
11	BPA	THF	85	2.5	3	3	Na	RT	3h	48	[f]
12	BPA	THF	85	1.5	4	5	Na	RT	overnight	100	multi
13	BPA	THF	85	1.5	4	8	Na	RT	overnight	100	multi
14	BPA	THF	85	1.5	6	5	Na	RT	overnight	96	mono
15	BPA	THF	170	1.5	4	5	Na	RT	overnight	100	mono
16	BPA [h]	THF	85	1.5	4	5	Na	RT	overnight	86	multi
17	BPA	DMF	170	1.5	4	5	Na	RT	overnight	100	multi
18	BPA	THF	170	3	8	10	DBU [g]	RT	5h	100	multi
19	BPN	THF	85	1.5	4	5	Na	RT	overnight	= [e]	[f]
20	BPN	THF	170	3	8	10	DBU [g]	RT	5h	100	multi
21	BPN	THF	170	1.5	4	5	Na	RT	overnight	= [e]	multi
22	BPN	THF	170	3	8	10	Na	RT	overnight	= [e]	multi
23	BPAM	THF	170	1.5	4	5	Na	RT	overnight	100	multi
24	BPAM	THF	170	3	8	10	Na	RT	overnight	100	multi
25	BPAM	THF	170	3	8	10	DBU [g]	RT	overnight	100	multi
26	BIB	DMF/H_2O	85	1.5	3	10	Na	50	6h	= [e]	[f]

27	BIB	DMF/H_2O	85	1.5	4	10	Na	70	6h	56	multi
28	BIB	DMF/H_2O	85	2.5	3	10	DBU[g]	70	6h	=[e]	multi
29	BIB	DMF/H_2O	85	1.5	4	10	Na	70	overnight	=[e]	[f]

[a] In some experiments, all reagents were doubled in concentration; these experiments are here highlighted as those with higher PS concentration, but please note that the PS / thiol stoichiometric ratio remains constant (=20) throughout all the table.

[b] Na was always the counterion during polymerization. In some cases, 2.2 equivalents of acetic acid were added at the end of the polymerization, followed by 2.3 equivalents of DBU, i.e. first thiolates and any residual alcoholate were protonated, then only thiols were deprotonated again with DBU leading to a large, organic counterion and a 'naked' thiolate.

[c] The initiator peak at 3.70 ppm in ^{1}H NMR was used as a reference to calculate the end-capping yield (mol%) from CH_2 of BB at 4.65 ppm; CH_3 of BP at 1.30 ppm, CH_3 of BPA at 1.30 ppm, CH of BPN at 3.50 ppm, CH of BPAM at 3.50 ppm, CH_3 groups of BIB overlapping with CH_3 of PPS at 1.30 ppm.

[d] The presence of multiple peaks is typically attributed to low end-capping yield, which allows thiolates to eventually produce disulfides, thereby multimerizing the macromolecular structure and generating multimodal molecular weight distributions.

[e] No resonance in the ^{1}H NMR spectrum can be associated to the end-capper structure.

[f] Not recorded, because it was already known that the end-capping performed poorly.

[g] DBU was always used in 1.15:1 molar ratio with thiols.

[h] In these experiments, 2.5 eq.s of azidobenzene (in relation to thiols) were added at the end of the PS polymerization and before the addition of CS_2, to avoid potential side reactions via quenching TBP.

1.4SI Characterization of the selected macroRAFT agents

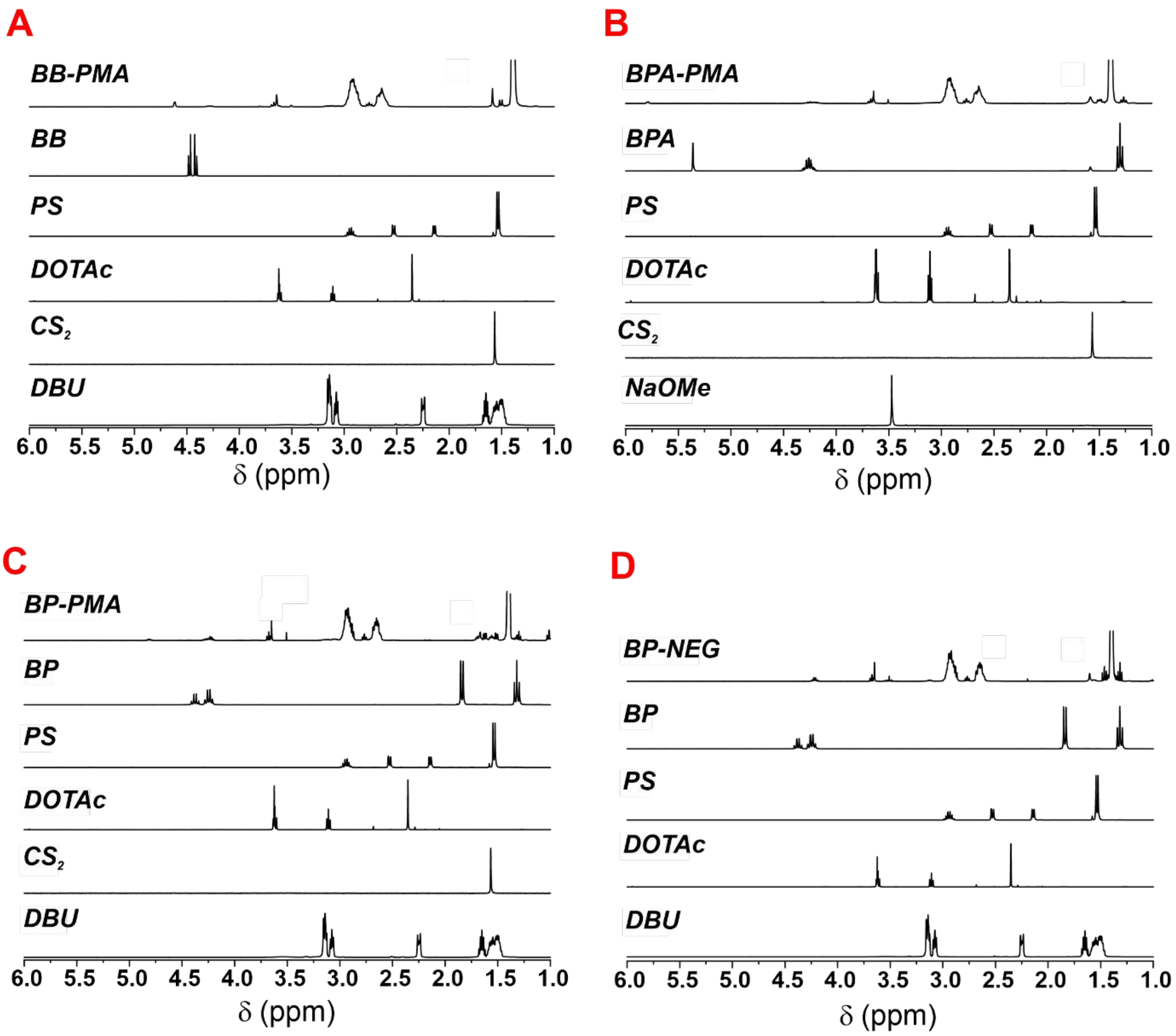

Figure S3. ^{1}H NMR spectra of the selected macroRAFT agents and their respective precursors. ***A.*** Benzyl bromide macroRAFT (BB-PMA); ***B.*** α-bromophenylacetate macroRAFT (BPA-PMA); ***C.*** 2-bromopropionate macroRAFT (BP-PMA); ***D.*** 2-bromopropionate without trithiocarbonate (BP-Neg). Other acronyms: Propylene sulfide (PS), dithiol acetate initiator (DOTAc), carbon disulfide (CS_2), 1,8-Diazabicyclo[5.4.0]undec-7-ene (DBU), sodium methoxide (NaOMe).

UV-spectroscopy was employed to quantify the number of C=S groups per chain, as well as to ensure the complete end-capping of the polymer chains. For that, a commercially available model molecule (bis(carboxymethyl)trithiocarbonate) was employed, measuring its extinction coefficient at 310 nm (ε=11,487 L/mol) and using the latter to calculate the molar concentation

of trithiocarbonates in THF solution. The molar concentration of the macromolecules in solution was obtained by dividing the polymer concentration in g/L by the $\overline{M_n}$ values obtained through GPC or MALDI-ToF.

According to the spectra presented in Figure S4, BP-PMA features the predicted two C=S groups per chain, but BB-PMA and BPA-PMA would appear to have around three. The result for the latter two, however, is difficult to rationalize on the basis of the synthetic approach: the three PMAs differ in the electrophile added in the last step, but the reaction leading to the trithiocarbonate (PPS tholates + CS_2) is identical; therefore, they should all have the same number of C=S groups. It is therefore likely that the extinction coefficient of aromatic-containing trithiocarbonates is simply different (higher) than that of a fully aliphatic one, i.e. bis(carboxymethyl)trithiocarbonate is an unsuitable model for PMA and BPA-PMA .

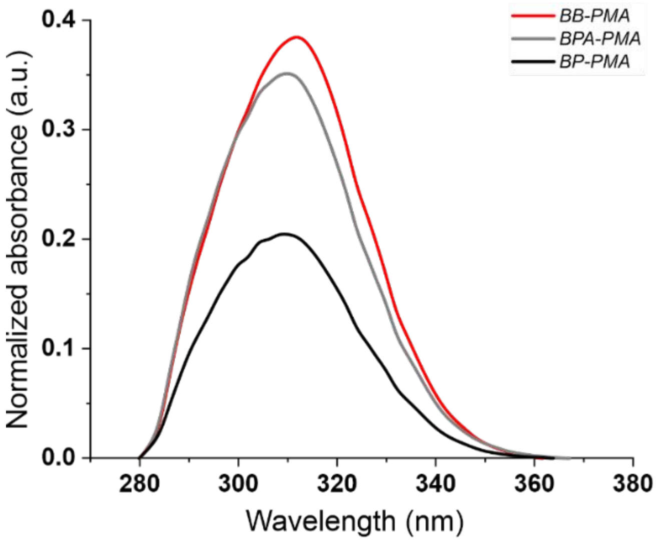

Figure S4. UV-spectra of the PPS-macroRAFT agents (PMAs). The absorbance values at 310 nm were used to calculate the concentration of C-S groups, according to Beer-Lambert's law. Please note that the peak location is the same despite the structural differences in the trithiocarbonate (a benzyl residue on BB-PMA (red), an aromatic β ester on BPA-PMA (light gray), and an aliphatic β ester on BP-PMA (black)), but the extinction coefficient of the band may and probably is affected by them.

1.5SI Optimization of MALDI-ToF analytical procedures

Our group previously reported MALDI-ToF analysis of PPS polymer end-capped with ethyl-2-bromoacetate using 2-(4-hydroxyphenylazo)benzoic acid (HABA) as matrix in the presence of silver trifluoroacetate (AgTFA) as a cationizing agent. Under the same conditions, however, no polymer signal acquisition was obtained with the polymers synthesized herein except for the BP-NEG, where the signal intensity was very low compared to the signals acquired at low-molecular weight region (Figure S5). We ascribed this to the different functionalities present at the polymer chain ends which might influence the ionization of the polymers. A more

hydrophobic matrix dithranol gave clearer polymer chain populations in the spectra where the best spectrum was obtained in the absence of a cationizing agent (Figure S6). These results confirmed that the polymer chain end group influences the ionization of a polymer. To identify the effect of trithiocarbonate at the polymer chain end, we performed a series of MALDI-ToF analysis of BP-PMA using different matrices, namely 1,4-dicyanobenzene (DCB), 2,5-dihydroxybenzoic acid (DHB), HABA, α-cyano-4-hydroxycinnamic acid (CHCA), or dithranol at varying polymer-to-matrix ratios in the presence or absence of NaTFA or AgTFA. Polymer chain population was observed only when HABA (only in the absence of a cationizing agent) or dithranol (except for the presence of AgTFA) was used. An increase in the polymer signal was observed when the polymer-to-matrix ratio was increased up to 1:19 and when linear ionization mode was employed but the fragment signals still overlapped with the polymer (Figure S7). Dithranol provided the best signal acquisition of BP-PMA with a negligible number of fragments in the spectrum when used in the absence of a cationizing agent and with relatively lower amount of matrix unlike HABA (Figures S8 and S9). The same was observed for BP-NEG (Figure S6) where the only difference between the chain end functionalities is the presence of trithiocarbonate group in BP-PMA. This indicates that the presence of trithiocarbonate does not significantly affect ionization of polymers.

We also investigated the chain end effect on the ionization of BB-PMA (Figures S10 and S11) and BPA-PMA (Figures S12 and S13) but only used HABA and dithranol since the polymer chain population was best observed when they were used in the previous trials of the other polymers. In the case of BB-PMA the best signal acquisition was recorded when HABA was used as a matrix in the presence of NaTFA. For BPA-PMA, both HABA&NaTFA and dithranol&no-salt conditions worked fine but the latter provided cleaner baseline with higher signal intensity of polymer chain population compared to the low-molecular weight fragments.

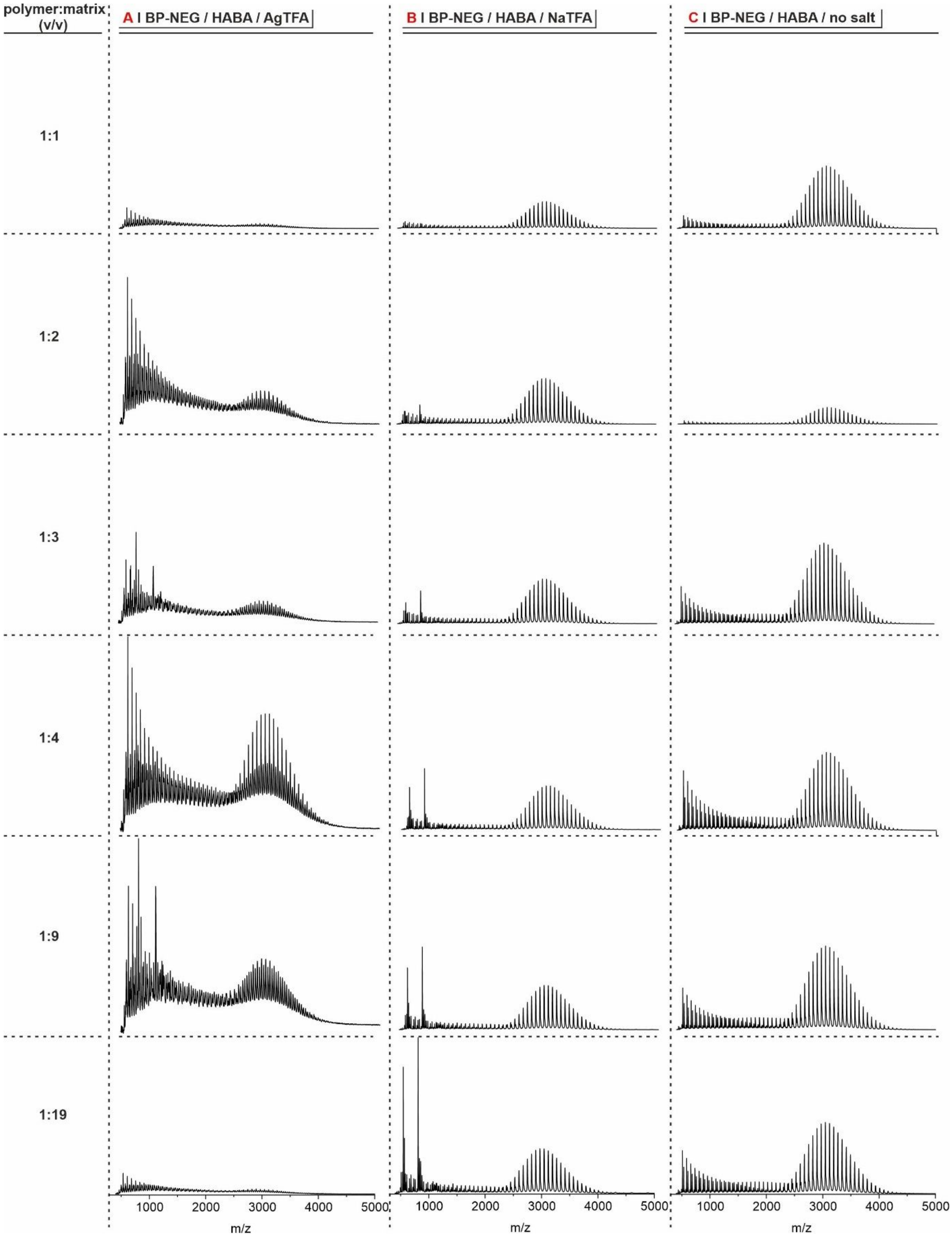

Figure S5. MALDI-ToF analysis of BP-NEG with HABA as a matrix in the presence or absence of cationizing agents at different polymer-to-matrix ratios (linear positive ion mode).

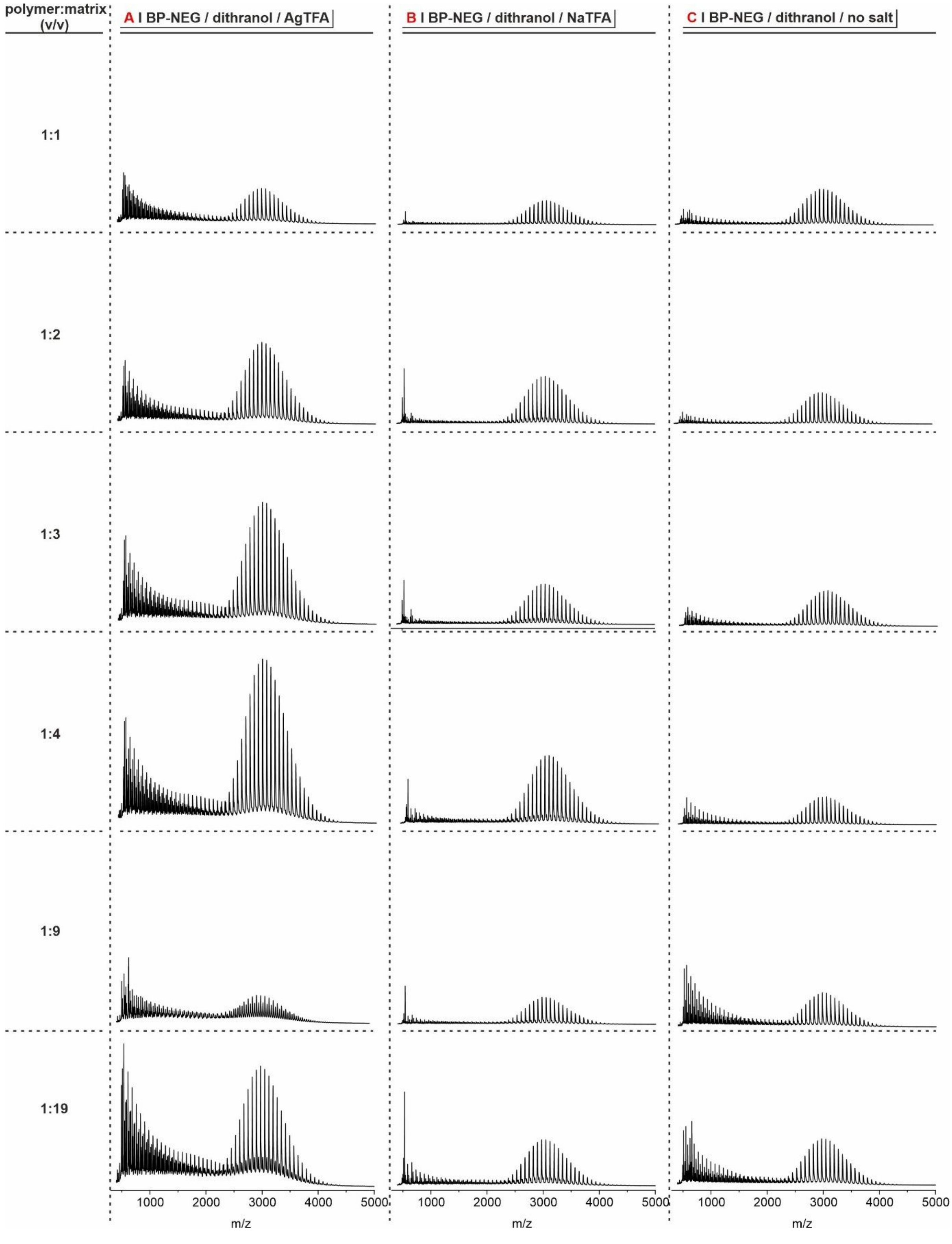

Figure S6. MALDI-ToF analysis of BP-NEG with dithranol as a matrix in the presence or absence of cationizing agents at different polymer-to-matrix ratios (linear positive ion mode).

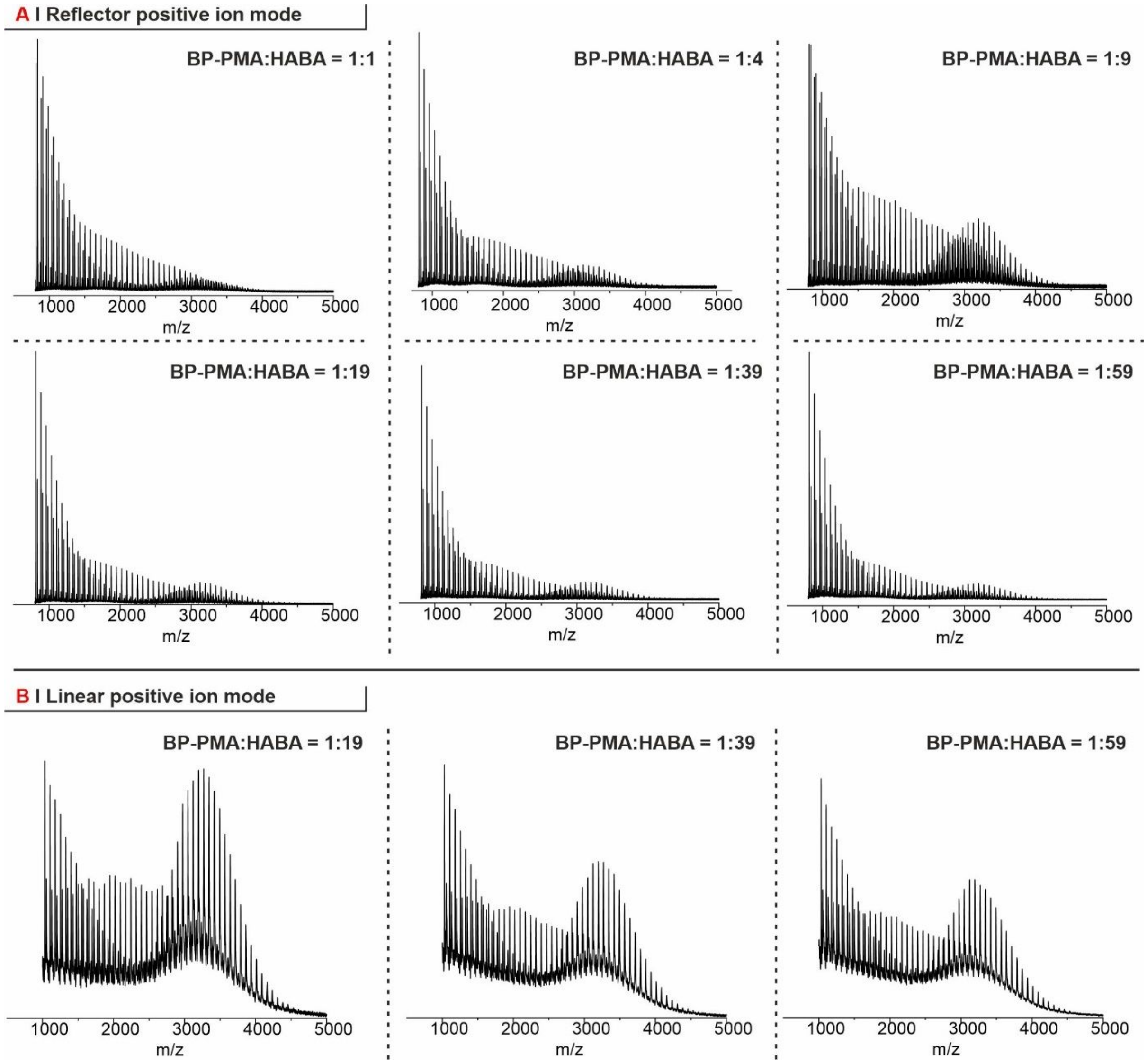

Figure S7. MALDI-ToF analysis of BP-PMA with HABA as a matrix in the absence of a cationizing agent at different polymer-to-matrix ratios.

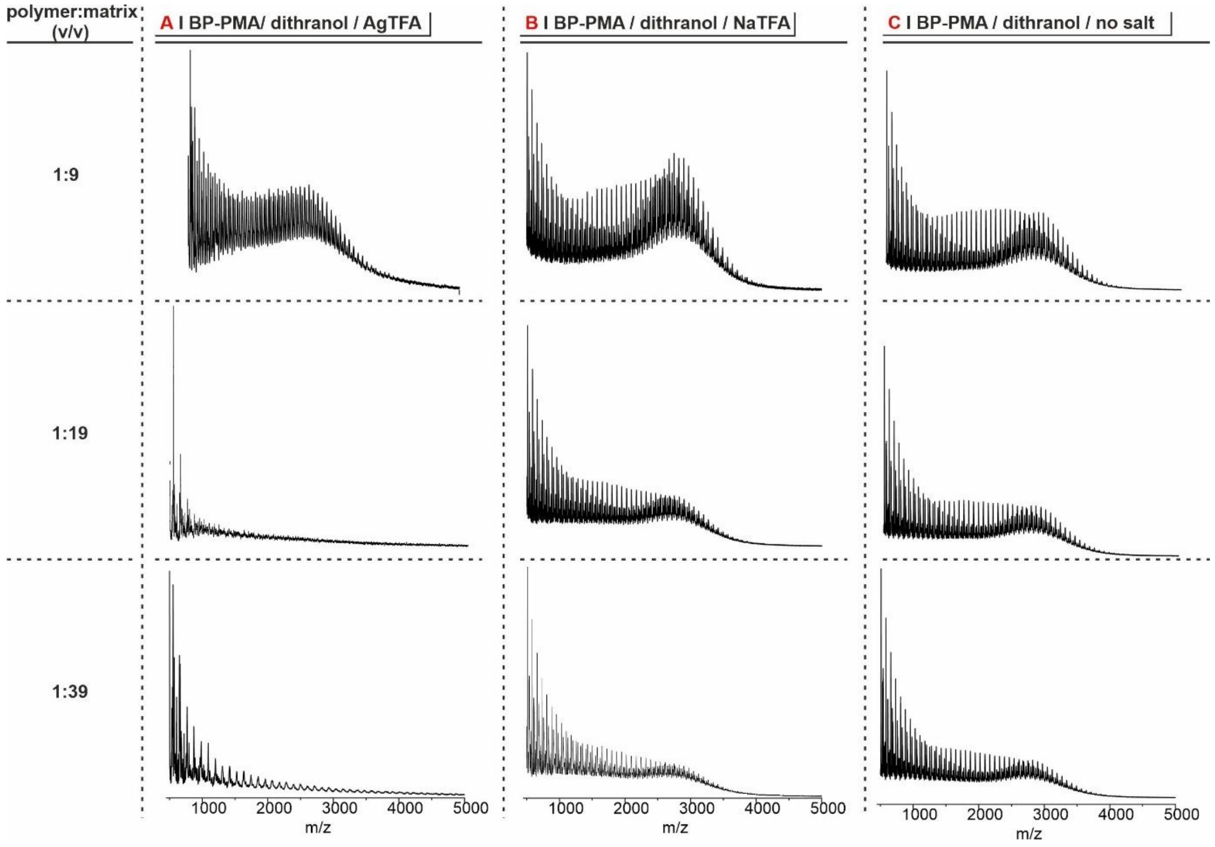

Figure S8. MALDI-ToF analysis of BP-PMA with dithranol as a matrix in the presence or absence of cationizing agents at different polymer-to-matrix ratios (linear positive ion mode).

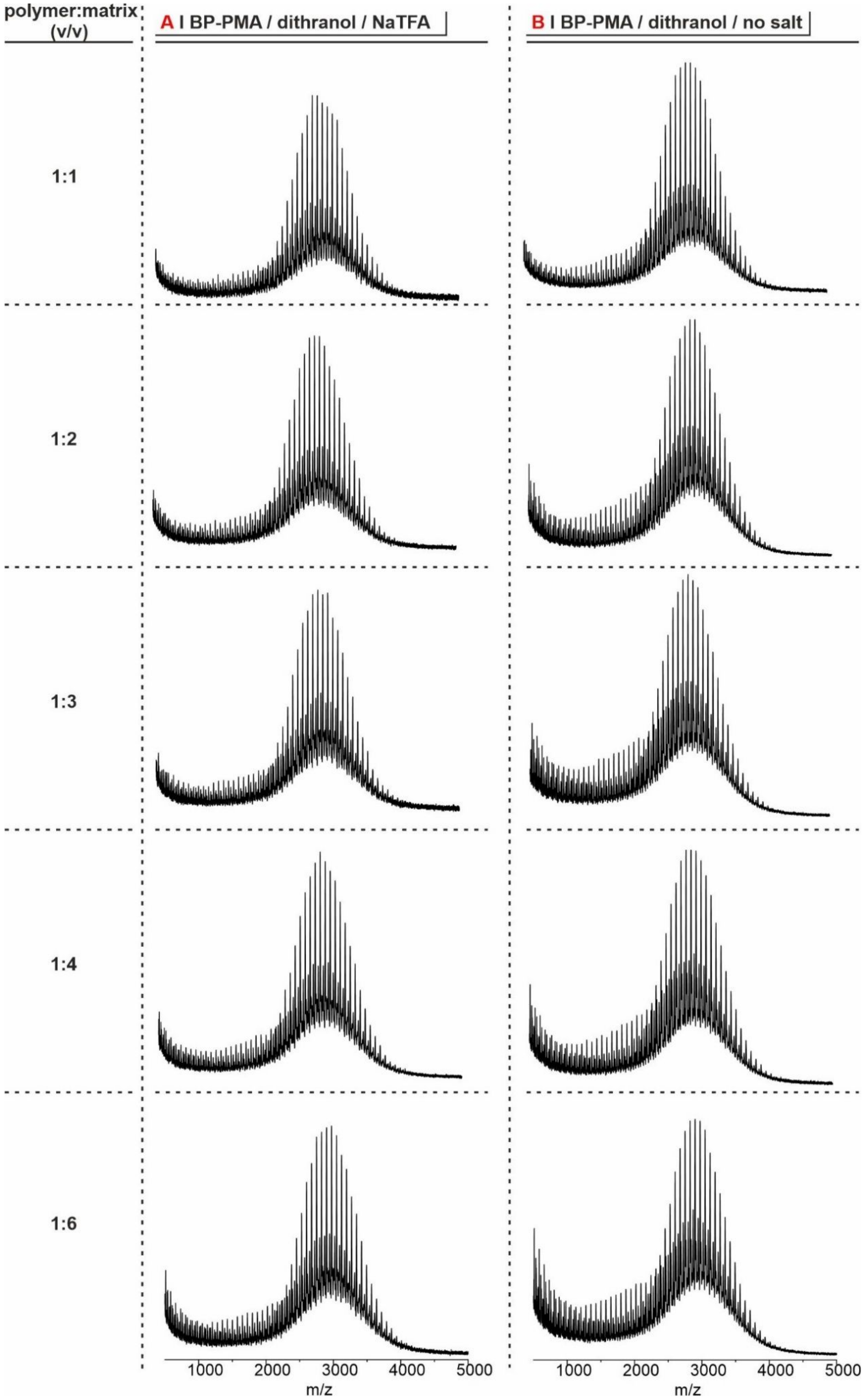
polymer:matrix (v/v)
A I BP-PMA / dithranol / NaTFA
B I BP-PMA / dithranol / no salt
1:1
1:2
1:3
1:4
1:6
1000
2000
3000
4000
5000
m/z

Figure S9. MALDI-ToF analysis of BP-PMA with dithranol as a matrix in the presence of NaTFA or without a cationizing agent at different polymer-to-matrix ratios (linear positive ion mode).

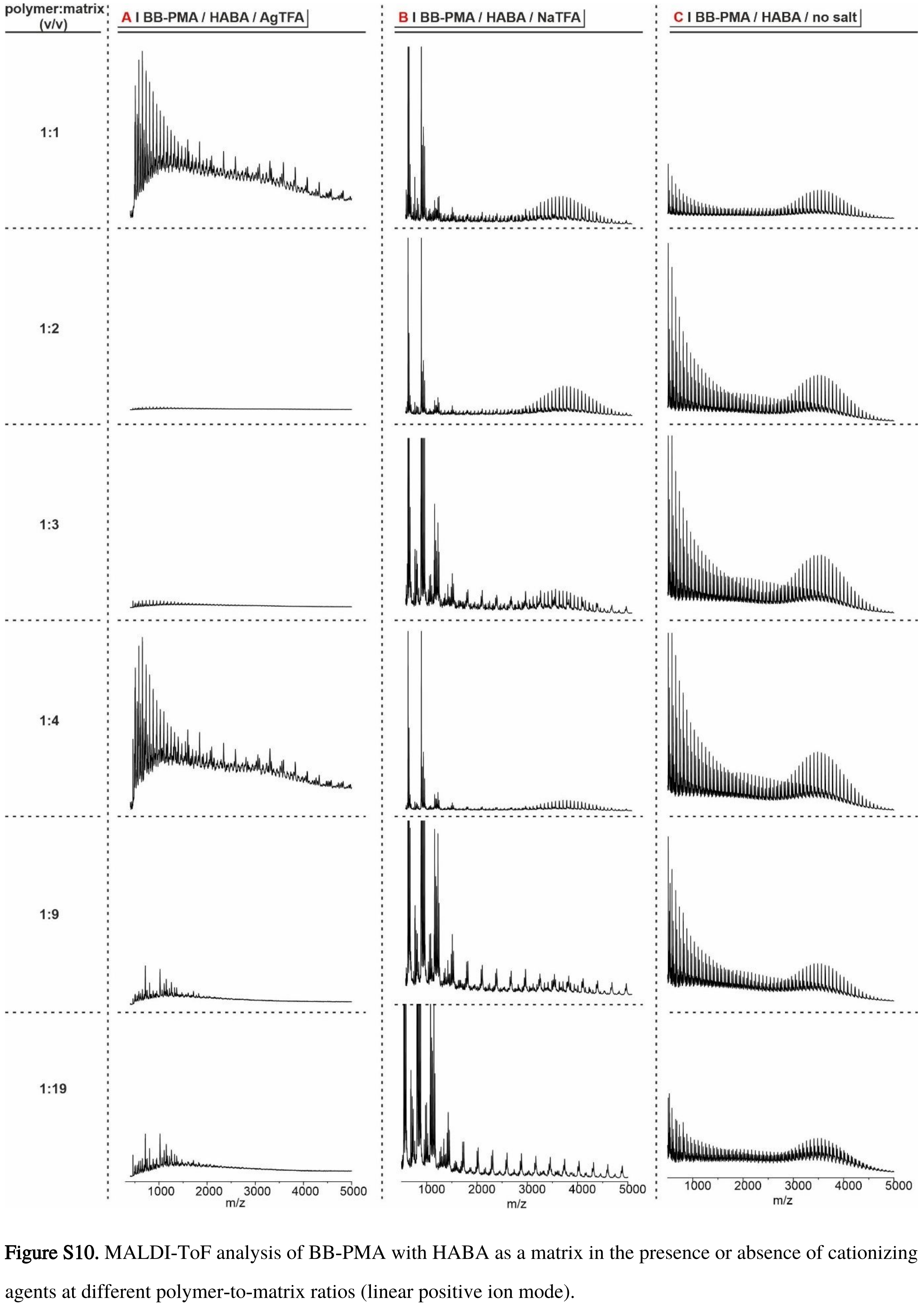

Figure S10. MALDI-ToF analysis of BB-PMA with HABA as a matrix in the presence or absence of cationizing agents at different polymer-to-matrix ratios (linear positive ion mode).

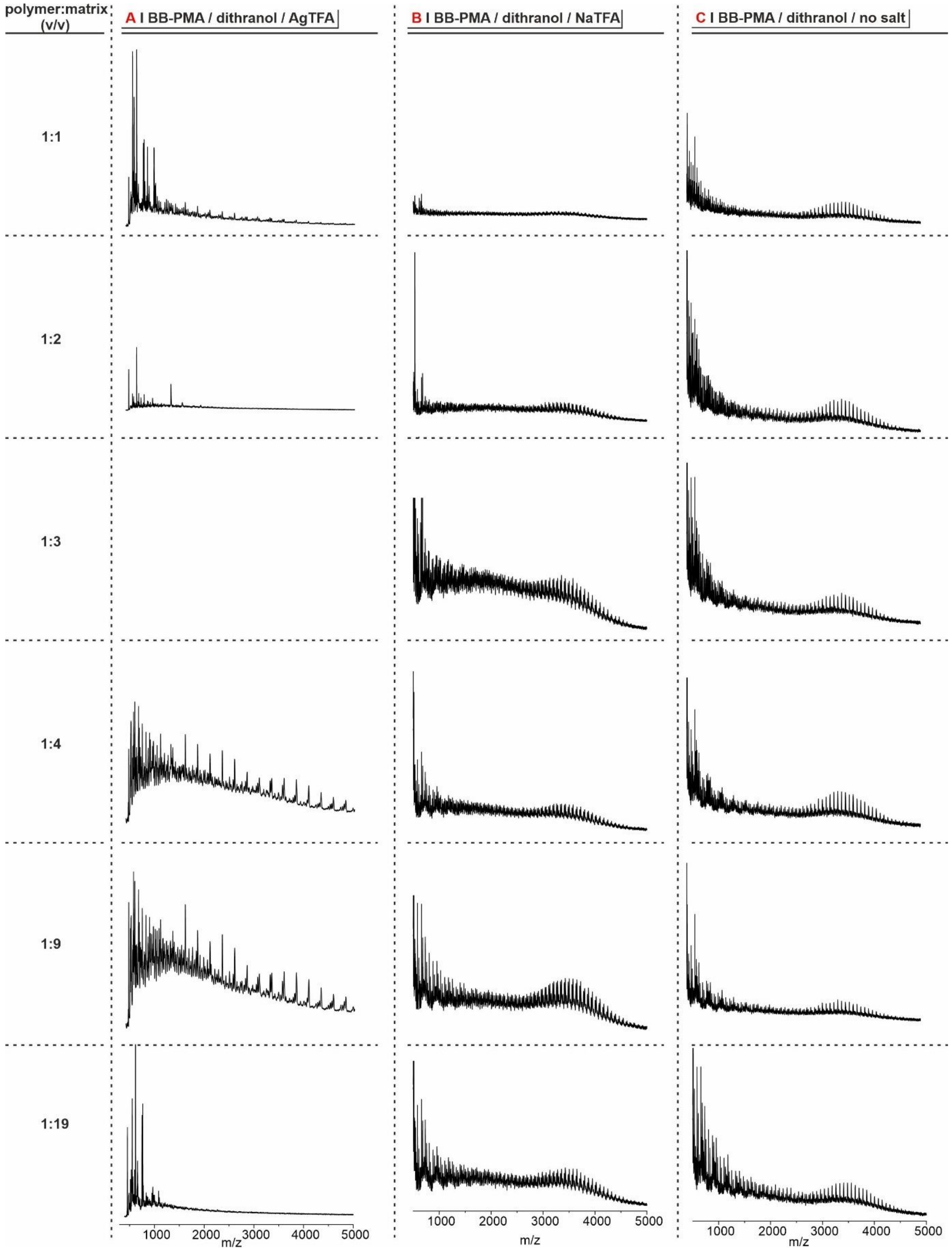

Figure S11. MALDI-ToF analysis of BB-PMA with dithranol as a matrix in the presence or absence of cationizing agents at different polymer-to-matrix ratios (linear positive ion mode).

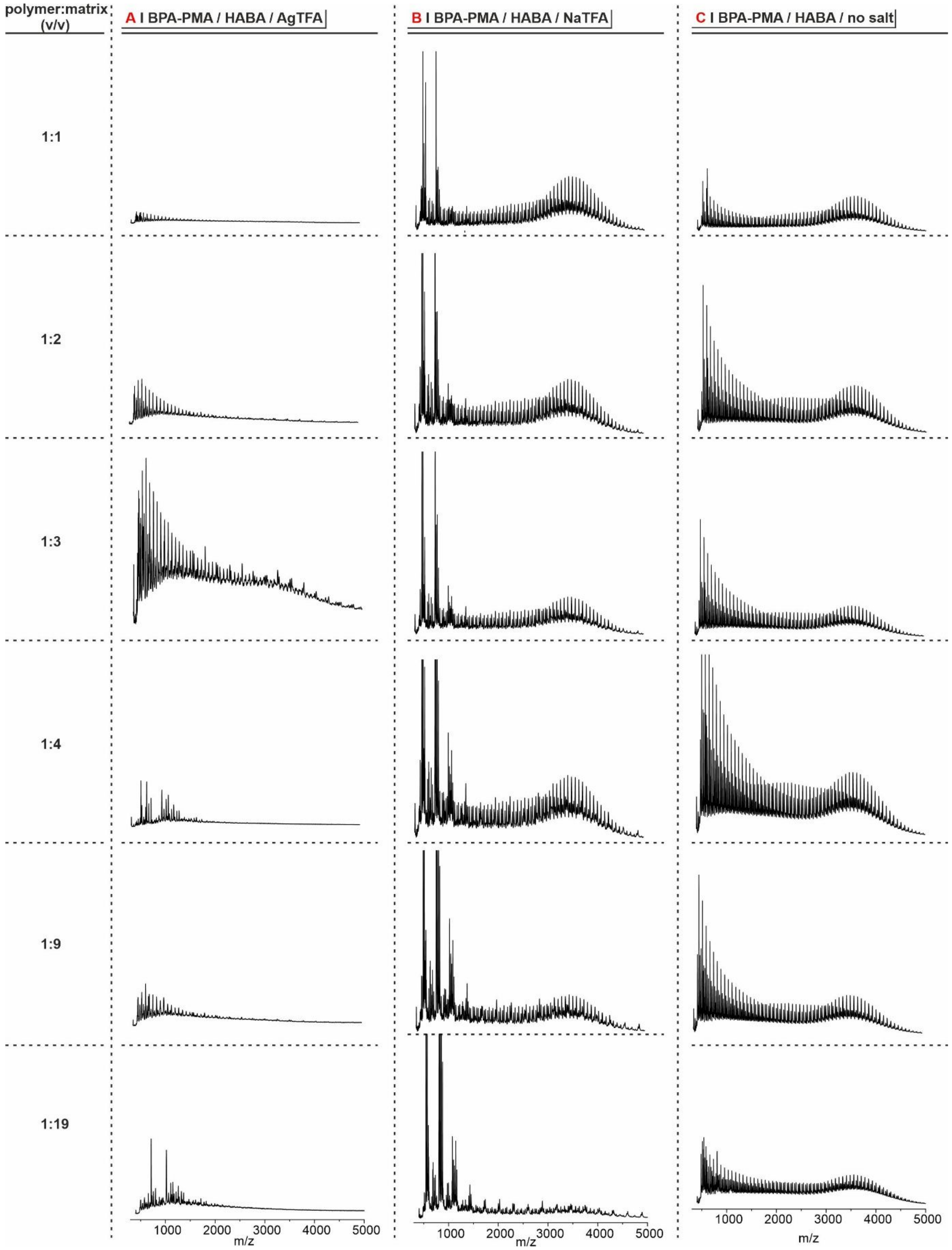

Figure S12. MALDI-ToF analysis of BPA-PMA with HABA as a matrix in the presence or absence of cationizing agents at different polymer-to-matrix ratios (linear positive ion mode).

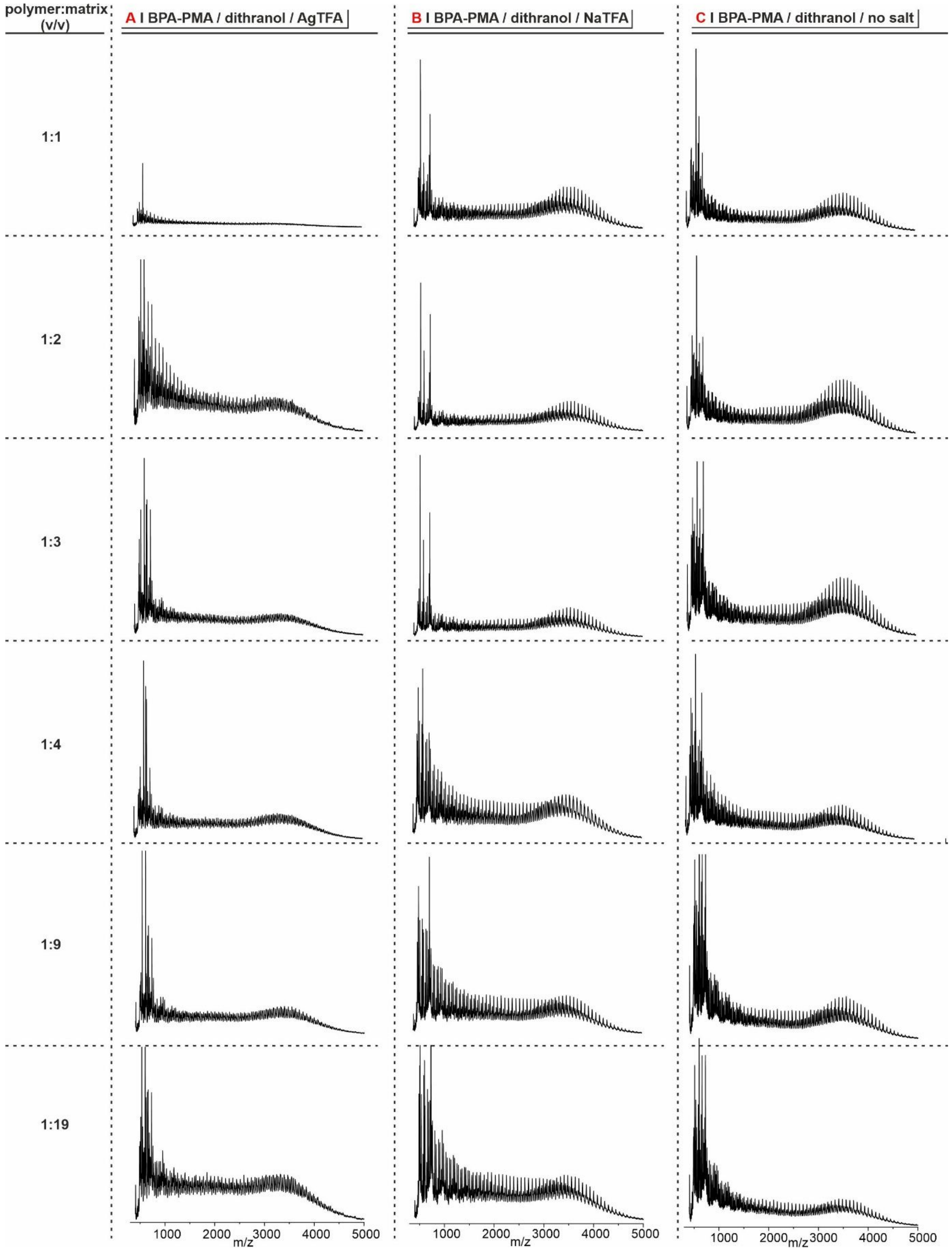

Figure S13. MALDI-ToF analysis of BPA-PMA with dithranol as a matrix in the presence or absence of cationizing agents at different polymer-to-matrix ratios (linear positive ion mode)

1.6SI RAFT polymerization of DMA/DEA

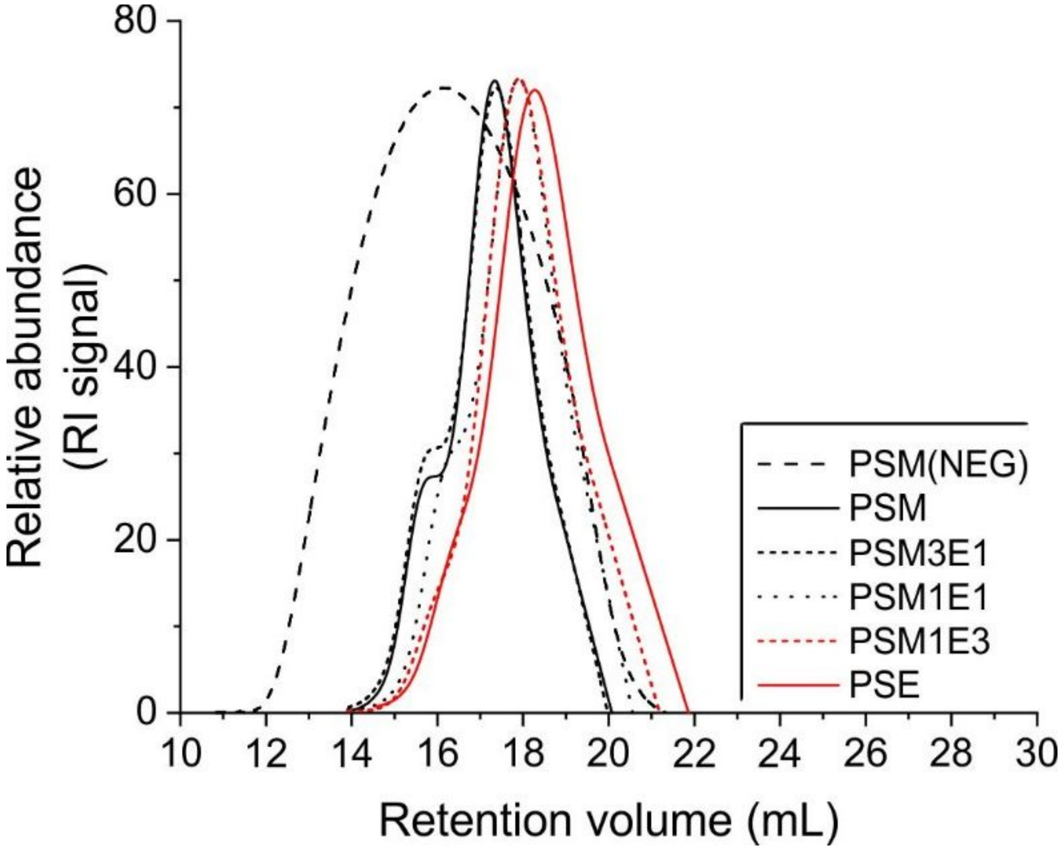

Figure S14. GPC traces of DMA/DEA homo- and copolymers synthesized using BP-PMA or the negative control BP-NEG. All samples were eluted in DMF containing 0.1% LiBr, 50 °C at a flow rate of 0.8 mL/min. Please note that a shoulder at low volumes (high molecular weight) is present proportionally to the DMA content in the monomer mixture. This shoulder accounts for about 7.5% of the polymer mass in PSM, progressively less for the others. We ascribe it to DMA being able to cleave some trithiocarbonates (the higher steric hindrance of DEA not making it possible), thereby uncovering one end of a thiol-terminated PPS; the latter can dimerize, producing a triblock structure with a longer PPS block. Due to the similarity in structure and the small amounts, it is assumed these materials not to affect the final properties of the material.

1.7SI Colloidal characterization

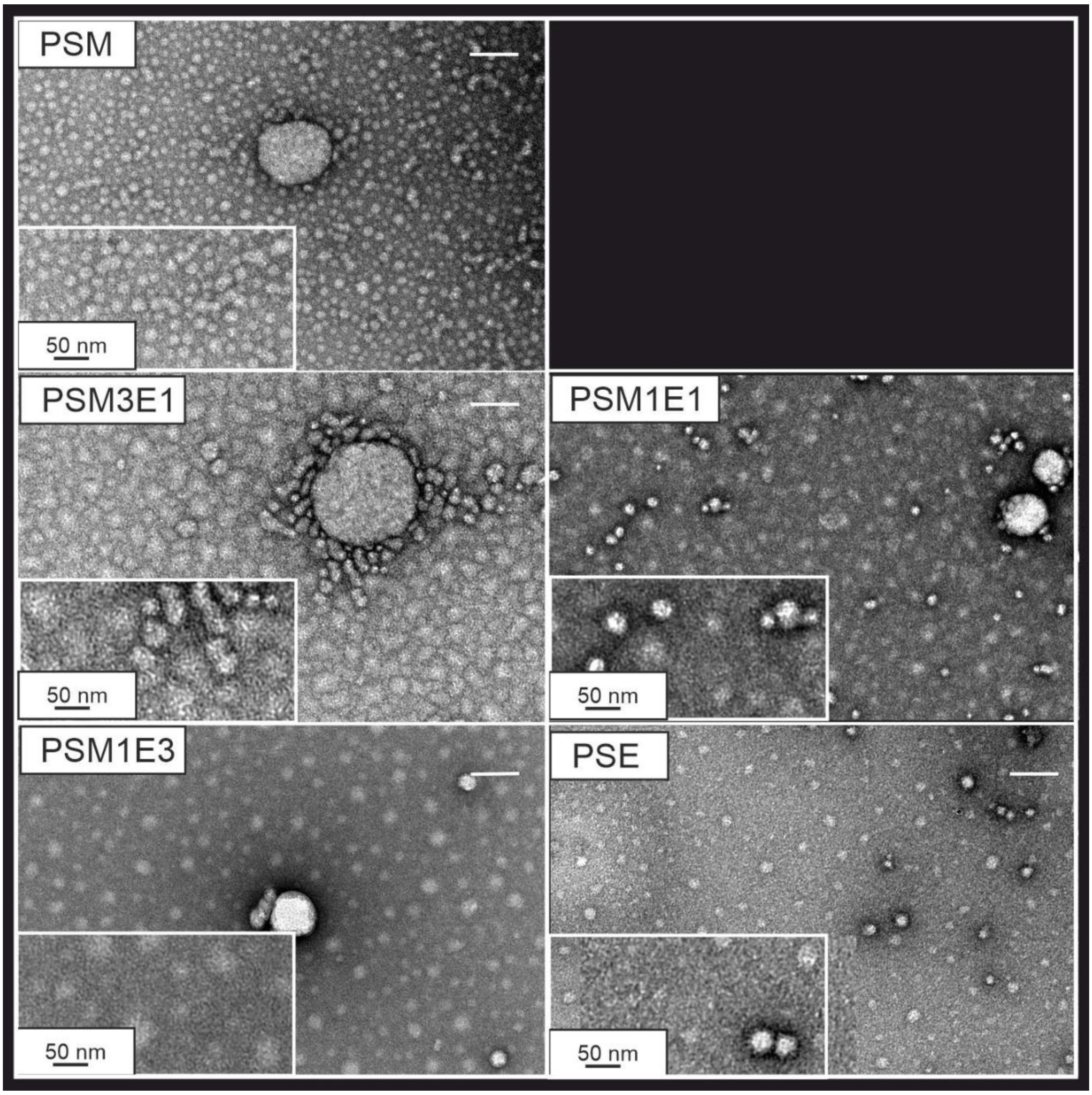

Figure S15. TEM images of aggregates of all block copolymers, obtained via rapid evaporation of water from 0.1 mg/mL suspensions in deionized water. Please note the presence of some large aggregates in almost all samples; micelles are the smaller-sized objects homogeneously distributed throughout the samples.

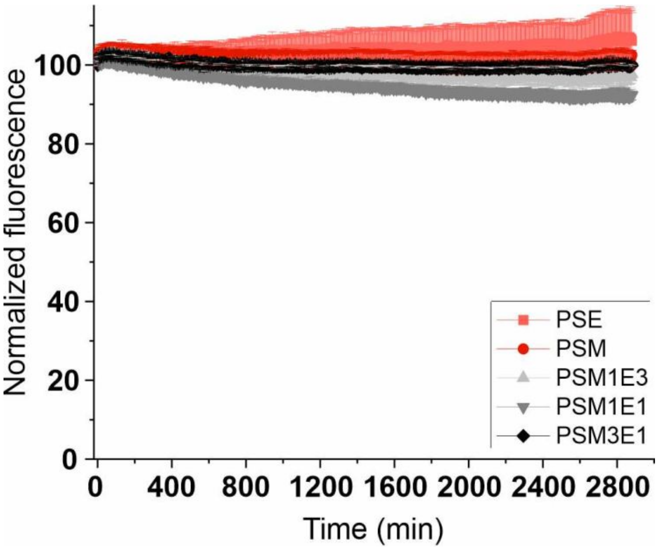

Figure S16. Nile Red fluorescence of the polymer aggregates in deionized water (4 mg/mL = 5 mM thioethers) in the absence of oxidants and as a function of time.

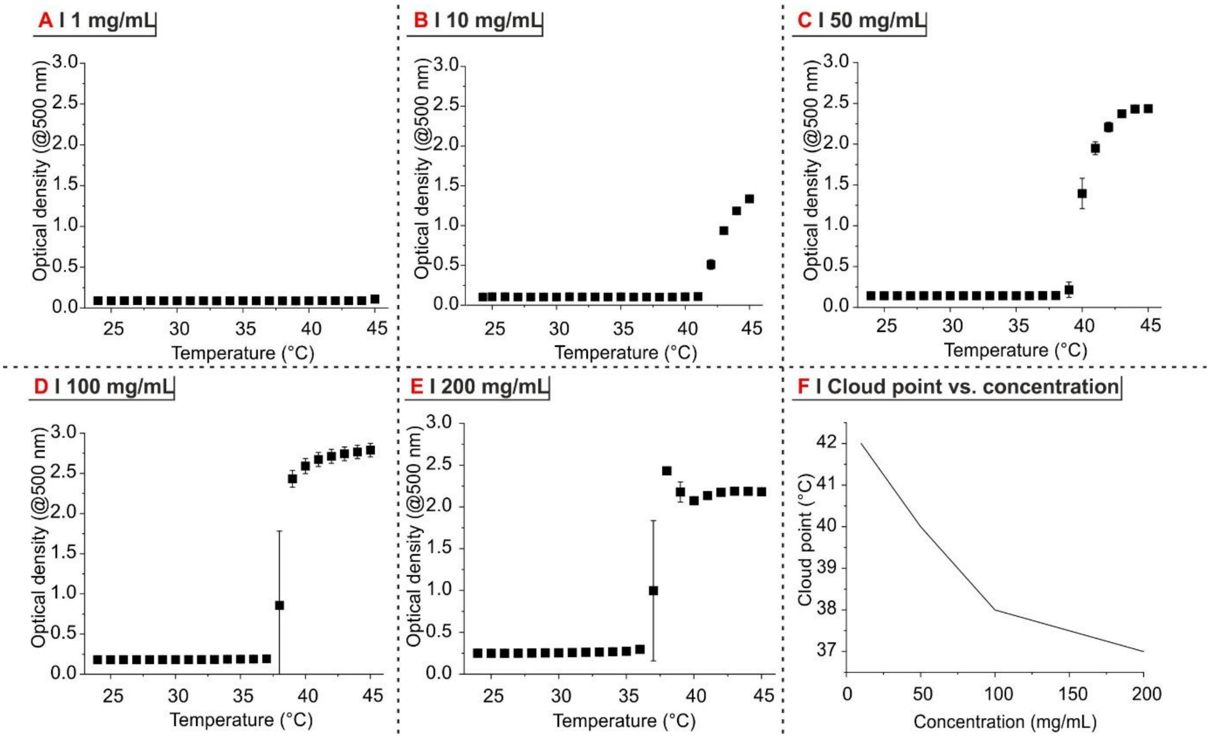

Figure S17. Dependency of the 500 nm optical density of PSE on temperature, at different concentrations in deionized water

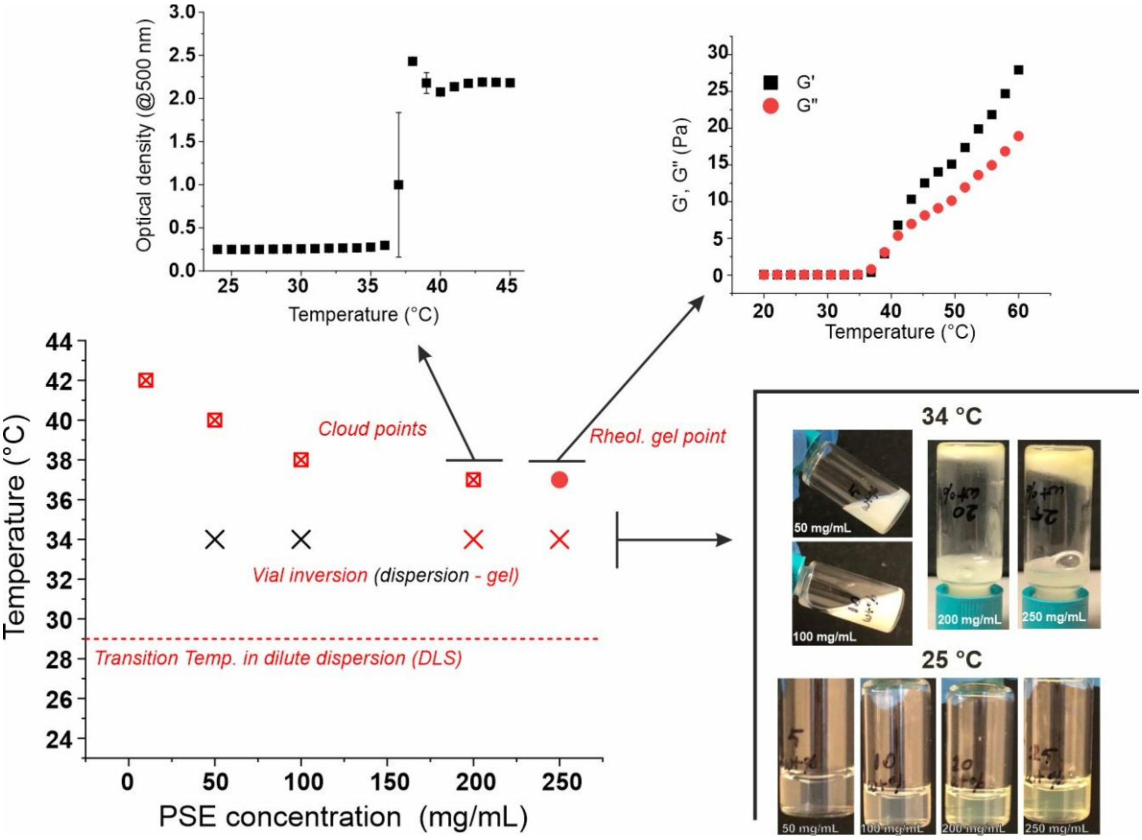

Figure S18. At the relatively low concentration of 1 mg/mL, PSE colloids aggregate into micron-size objects at about 29˚C (dashed red line). Cloud points are recorded as the temperatures where the 500 nm optical density of more concentrated, 10-200 mg/mL PSE dispersions show a steep increase (inset in the top left of the figure; the turbidity scans for other concentrations are reported Figure S17); these transitions correspond to the formation of much larger aggregates and therefore occur at higher temperatures then what observed at 1 mg/mL. At higher concentrations, the dispersions scatter too much already at room temperature to allow cloud points to be detected, but it is remarkable that the cloud point recorded at 200 mg/mL (around 37˚C) coincides with the gel point provided by shear rheology (inset in the top right of the figure). It is further noted that vial inversion tests performed at 34˚C showed that 200 and 250 mg/mL dispersions are almost completely gelled.

1.8SI Biological characterization

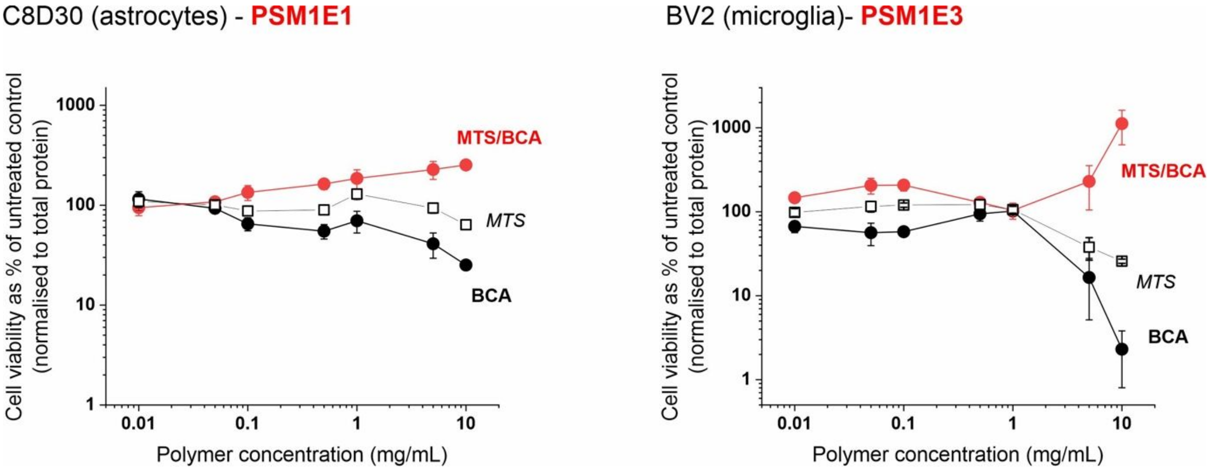

Figure S19. Two examples of the use of 'simple' MTS assay (empty squares) in comparison to the combination of MTS 'per cell' (MTS/BCA, red circles) with number of attached cells (BCA, black squares). The latter two parameters amplify the intensity of the effects and allow a better appreciation of the polymer concentration above which potentially toxic effects may arise.

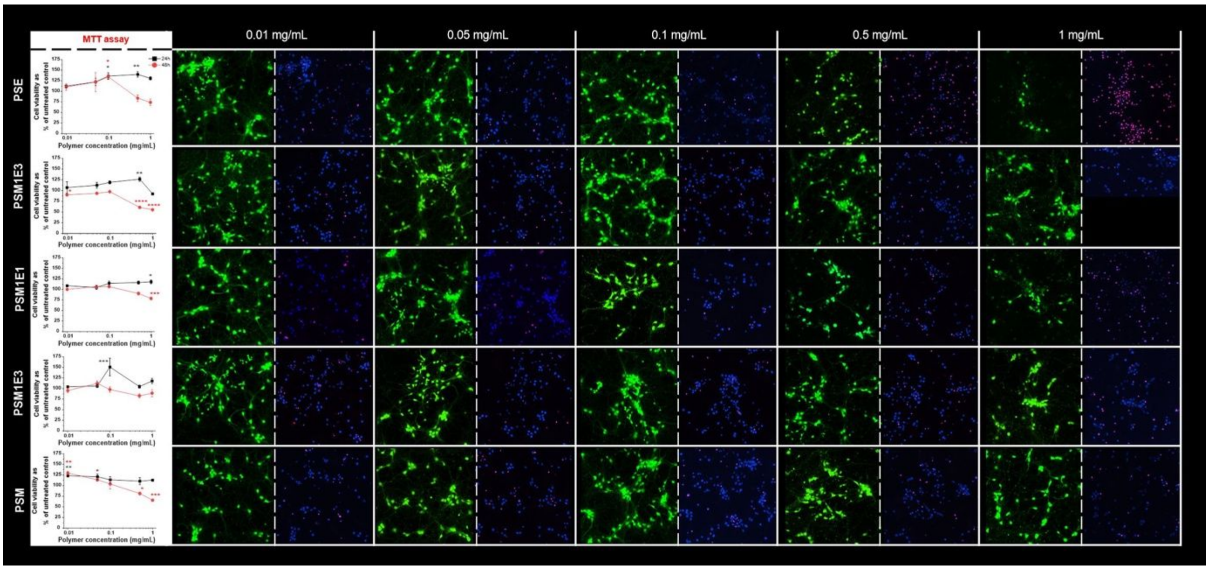

Figure S20. *Left.* The 48h exposure of neurons to all polymers measurably decreased the viability in comparison to 24 h, although it only approached 50% of the MTT signal (not normalized against protein content) at the highest concentrations (≥ 0.5 mg/mL) of the polymers with the largest DEA content (PSE and PSM1E3). *Right.* Live-dead stains (calcein / propidium iodide) con cortical neurons exposed for 24h to all polymers at five different concentrations.
